# Supplementary material for: Modelling the impact of JNJ-1802, a first-in-class dengue inhibitor blocking the NS3-NS4B interaction, on in-vitro DENV-2 dynamics
Source: PLoS Comput Biol. 2023 Dec 6;19(12):e1011662. doi: 10.1371/journal.pcbi.1011662 (PMC10699615; doi:10.1371/journal.pcbi.1011662)
Supplement: S1 Text — Fig A. Variation in viral RNA measurements. Co-efficient of variation (CV) for the log10 viral RNA measurement obtained from experimental infection studies in Vero cells, disaggregated by measurement type, JNJ-1802 concentration and DENV-2 strain. CV is the ratio of the standard deviation to the mean. Fig B. Model Fits (DENV-2/16681 strain, with refresh). Model fits for the measurements observed using the DENV-2/16681 strain with medium refresh on day 4 and antiviral concentrations of 0 nM, 2.56x10^-03^ nM, 1.28x10^-02^ nM, 6.40x10^-02^ nM, 0.32 nM, 1.6 nM, and 8 nM. Coloured points represent the data from each well (6 wells for 0 nM, 4 wells for concentrations >0 nM), the vertical red line indicates the time the viral inoculum was added to each well and the horizontal dashed black line indicates the limit of detection. The modelled dynamics of the intracellular RNA virus are in green and those of the extracellular RNA virus are in purple; solid lines represent the median and the shading represents the 95% CrI. Viral suppression is observed for concentrations 1.6 nM and 8 nM. Here, measurements below the limit of quantification (crosses in Fig 1) were included during model fitting. Measurements below the LOD were left censored at the LOD during model fitting and were plotted at the LOD for visual display. We assumed that the antiviral directly inhibits the transition process of infected cells to infectious (virion producing) cells, i.e., acts on τ. Fig C. Model Fits (DENV-2/RL strain, with refresh). Model fits for the measurements observed using the DENV-2/RL strain with medium refresh on day 4 and antiviral concentrations of 0 nM, 2.56x10^-03^ nM, 1.28x10^-02^ nM, 6.40x10^-02^ nM, 0.32 nM, 1.6 nM, and 8 nM. Coloured points represent the data from each well (6 wells for 0nM, 4 wells for concentrations >0nM), the vertical red line indicates the time the viral inoculum was added to each well and the horizontal dashed black line indicates the limit of detection. The modelled dynam [file pcbi.1011662.s002.pdf]

S1 Text: Supplementary Material

Modelling the impact of JNJ-1802, a first-in-class dengue inhibitor blocking the NS3-NS4B interaction, on in-vitro DENV-2 dynamics

Clare P. McCormack[1*], Olivia Goethals[2], Nele Goeyvaerts[3], Xavier D. Woot de Trixhe[3], Peggy Geluykens[3], Doortje Borrenberghs[2], Neil M. Ferguson[1], Oliver Ackaert[3+*], Ilaria Dorigatti[1+]

[1]MRC Centre for Global Infectious Disease Analysis, School of Public Health, Imperial College London, London, United Kingdom

[2] Janssen Global Public Health, Janssen Pharmaceutica NV, Beerse, Belgium

[3] Janssen Research & Development, Janssen Pharmaceutica NV, Beerse, Belgium

[*] c.mccormack14@imperial.ac.uk (CPM) oackaert@ITS.JNJ.com (OA)

[+] joint senior authors

Table of Contents

Table of Contents 2
1 RNA Measurements 3
2 Model Fits 4
2.1 Impact of Medium Refresh 4
2.2 Cell Dynamics 8
2.3 Goodness of Fit 10
2.4 Limit of Quantification 11
3 Alternative Mode of Drug Action 15
3.1 Mathematical Model 15
3.2 Model Fits 19
4 Sensitivity to Viral Inoculum 25
5 Sensitivity to Data Excluded 27
6 Deriving the basic reproduction number 29

1 RNA Measurements

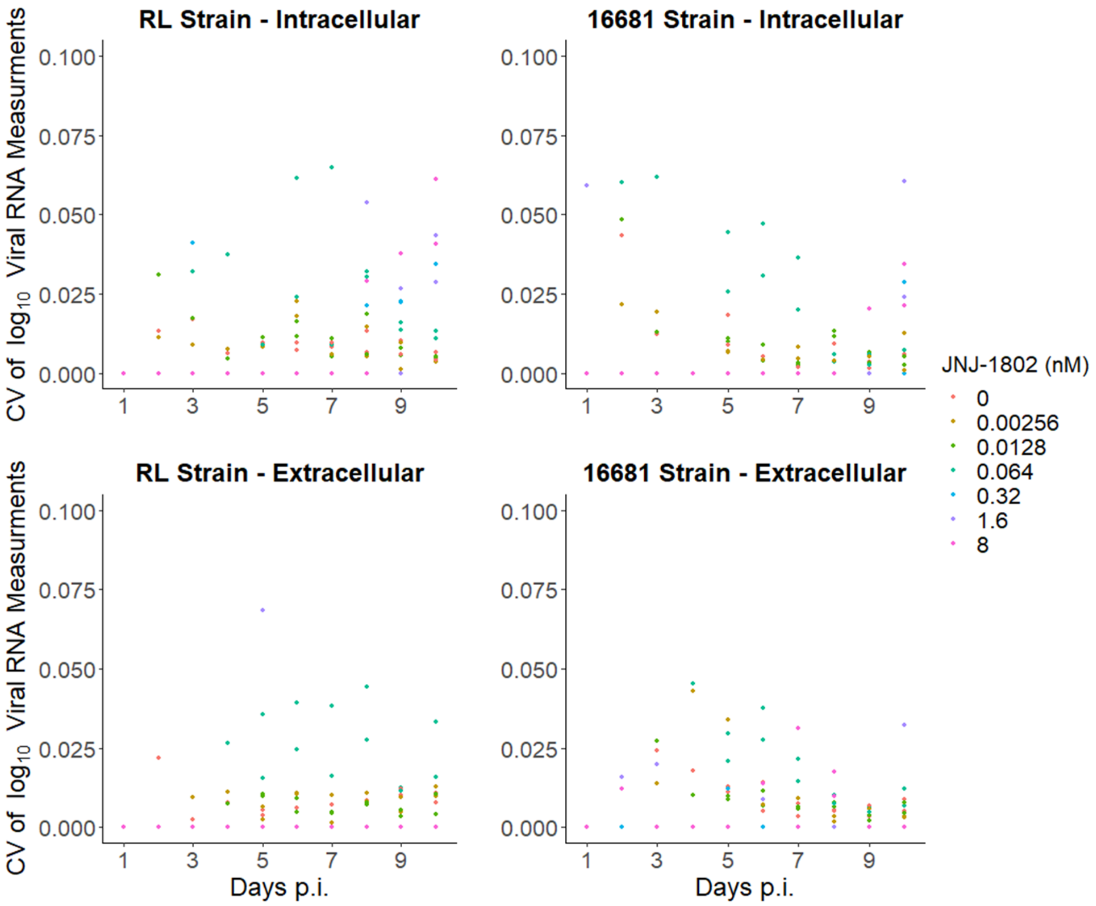

Fig A: Variation in viral RNA measurements. Co-efficient of variation (CV) for the $\log_{10}$ viral RNA measurement obtained from experimental infection studies in Vero cells, disaggregated by measurement type, JNJ-1802 concentration and DENV-2 strain. CV is the ratio of the standard deviation to the mean.

2 Model Fits

2.1 Impact of Medium Refresh

		DENV-2/RL		DENV-2/16681	
**Parameter**	**Description**	**No Refresh**	**With Refresh**	**No Refresh**	**With Refresh**
**β**	Infection rate of target cells per virion (day^{-1})	3.18×10^{-08} (2.29×10^{-08},4.40×10^{-08})	3.23×10^{-08} (2.27×10^{-08},4.72×10^{-08})	1.32×10^{-08} (9.05×10^{-09},1.88×10^{-08})	1.46×10^{-08} (1.00×10^{-08},2.10×10^{-08})
**ω**	Intracellular virus production rate per infectious cell (day^{-1})	3.17×10^{6} (2.32×10^{6},4.44×10^{6})	5.05×10^{6} (3.57×10^{6},7.16×10^{6})	1.36×10^{7} (9.43×10^{6},1.89×10^{7})	1.55×10^{7} (1.10×10^{7},2.36×10^{7})
**p**	Proportion of intracellular RNA becoming extracellular virus	5.90×10^{-01} (3.99×10^{-01},8.83×10^{-01})	3.78×10^{-01} (2.44×10^{-01},5.59×10^{-01})	2.28×10^{-01} (1.55×10^{-01},3.55×10^{-01})	1.87×10^{-01} (1.22×10^{-01},2.79×10^{-01})
**IC_{50}**	Concentration at which 50% of maximum effect is achieved (nM)	1.23×10^{-02} (8.33×10^{-03},1.66×10^{-02})	6.71×10^{-03} (4.07×10^{-03},1.03×10^{-02})	1.28×10^{-02} (9.00×10^{-03},1.79×10^{-02})	6.90×10^{-03} (4.45×10^{-03},9.45×10^{-03})
**h**	Hill coefficient	1.28 (1.14,1.42)	0.98 (0.89,1.09)	1.17 (1.07,1.31)	0.92 (0.85,1.00)
**R_0**	Basic reproduction number	277.01 (231.78,332.66)	286.37 (233.24,360.98)	191.10 (161.84,232.76)	203.39 (169.74,241.67)
**σ_V**	Residual error standard deviation (extracellular measurements)	0.83 (0.78,0.88)			
**σ_X**	Residual error standard deviation (intracellular measurements)	0.91 (0.86,0.97)			
**L**	Log-likelihood	-1619.87 (-1627.66,-1614.25)			
**DIC**	Deviance Information Criterion	3,254			

Table A: Posterior parameter estimates. Median posterior estimate and 95% credible interval (CrI) in brackets. Here the measurements below the limit of quantification (crosses in Figure 1) were included during model fitting, and we assumed that the antiviral directly inhibits transition of infected cells to infectious (virion producing) cells, i.e., acts on τ.

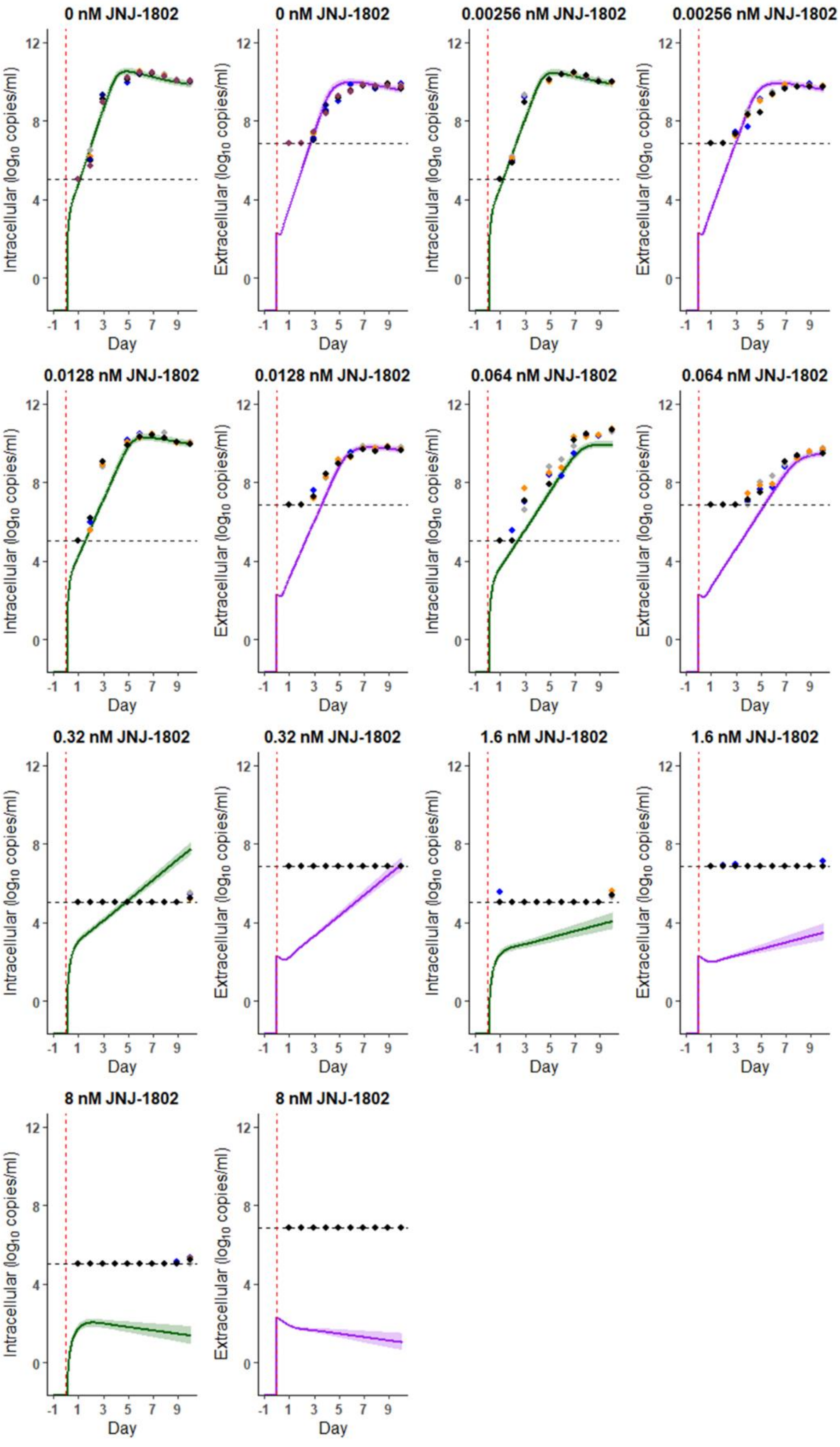

Fig B: Model Fits (DENV-2/16681 strain, with refresh). Model fits for the measurements observed using the DENV-2/16681 strain with medium refresh on day 4 and antiviral concentrations of 0 nM, 2.56x10^{-03} nM, 1.28x10^{-02} nM, 6.40x10^{-02} nM, 0.32 nM, 1.6 nM, and 8 nM. Coloured points represent the data from each well (6 wells for 0 nM, 4 wells for concentrations >0 nM), the vertical red line indicates the time the viral inoculum was added to each well and the horizontal dashed black line indicates the limit of detection. The modelled dynamics of the intracellular RNA virus are in green and those of the extracellular RNA virus are in purple; solid lines represent the median and the shading represents the 95% CrI. Viral suppression is observed for concentrations 1.6 nM and 8 nM. Here, measurements below the limit of quantification (crosses in Figure 1) were included during model fitting. Measurements below the LOD were left censored at the LOD during model fitting and were plotted at the LOD for visual display. We assumed that the antiviral directly inhibits the transition process of infected cells to infectious (virion producing) cells , i.e., acts on τ .

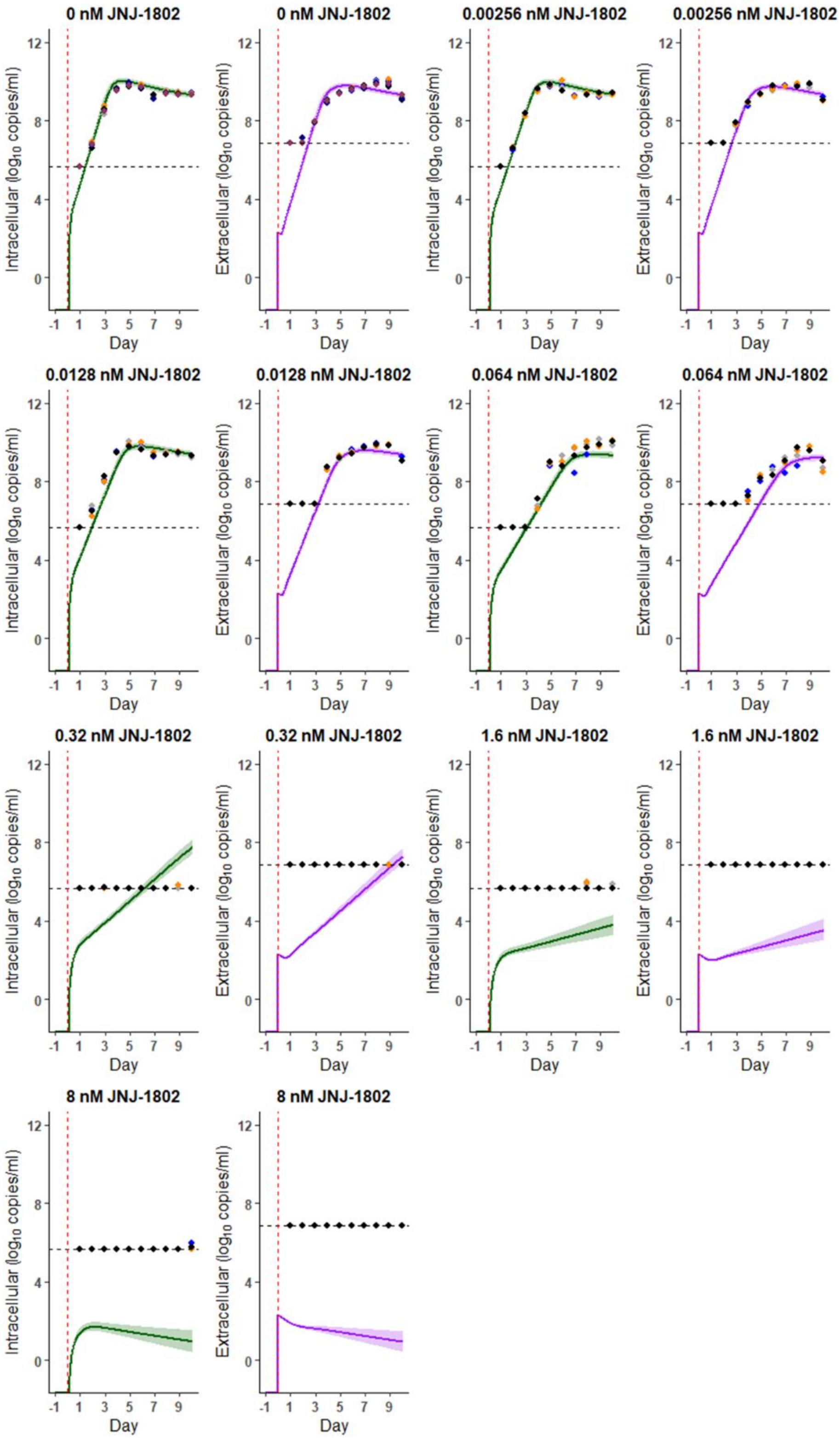

Fig C: Model Fits (DENV-2/RL strain, with refresh). Model fits for the measurements observed using the DENV-2/RL strain with medium refresh on day 4 and antiviral concentrations of 0 nM, 2.56×10^{-03} nM, 1.28×10^{-02} nM, 6.40×10^{-02} nM, 0.32 nM, 1.6 nM, and 8 nM. Coloured points represent the data from each well (6 wells for 0nM, 4 wells for concentrations >0nM), the vertical red line indicates the time the viral inoculum was added to each well and the horizontal dashed black line indicates the limit of detection. The modelled dynamics of the intracellular RNA virus are in green and those of the extracellular RNA virus are in purple; solid lines represent the median and the shading represents the 95% CrI. Viral suppression is observed for concentrations 1.6 nM and 8 nM. Here, measurements below the limit of quantification (crosses in Figure 1) were included during model fitting. Measurements below the LOD were left censored at the LOD during model fitting and were plotted at the LOD for visual display. We assumed that the antiviral directly inhibits the transition process of infected cells to infectious (virion producing) cells , i.e., acts on τ .

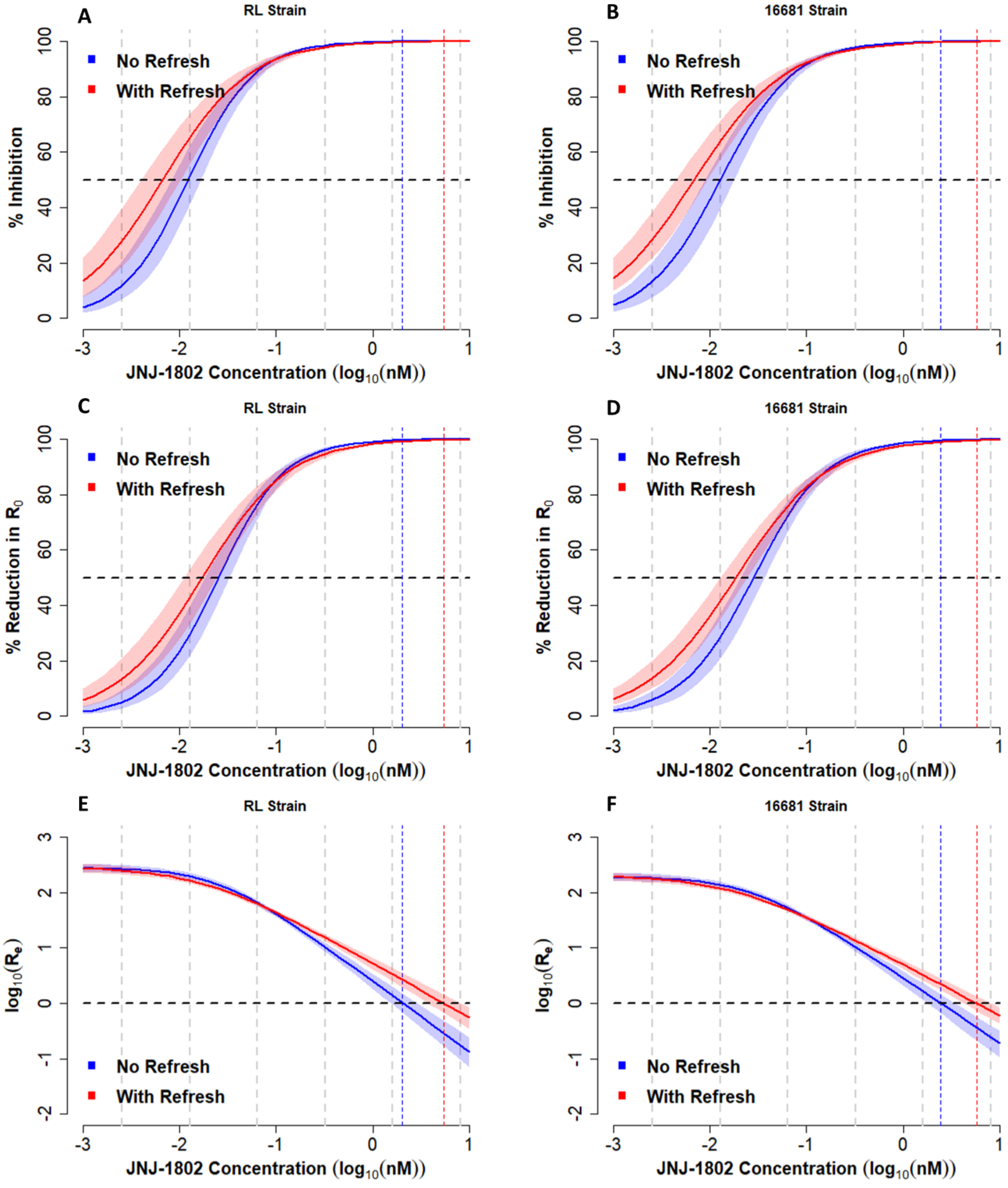

Fig D: Impact of Medium Refresh. Estimated inhibition percentage (A,B), percentage reduction in the basic reproduction number R_0 (C,D) and effective reproduction number R_e (E,F) as a function of concentration, for the DENV-2/RL strain (A, C, E) and DENV-2/16681 strain (B, D, F) where the well medium was refreshed or not on day 4. Estimates were calculated by substituting 1,000 parameter values sampled from the posterior distribution into equations (1), (6) and (7) in the main text. Solid lines represent the median, the shading represents the 95% CrI, dotted grey vertical lines indicate the concentrations tested in the in vitro experiments, and the dotted blue and red vertical lines indicate the median concentration such the R_e =1. The dotted black horizontal lines indicate the threshold for a 50% reduction (A,B,C,D), and when R_e =1 (E,F). Here, measurements below the limit of quantification (crosses in Figure 1) were included during model fitting and we assume the antiviral directly inhibits the transition process of infected cells to infectious (virion producing) cells, i.e., acts on τ .

2.2 Cell Dynamics

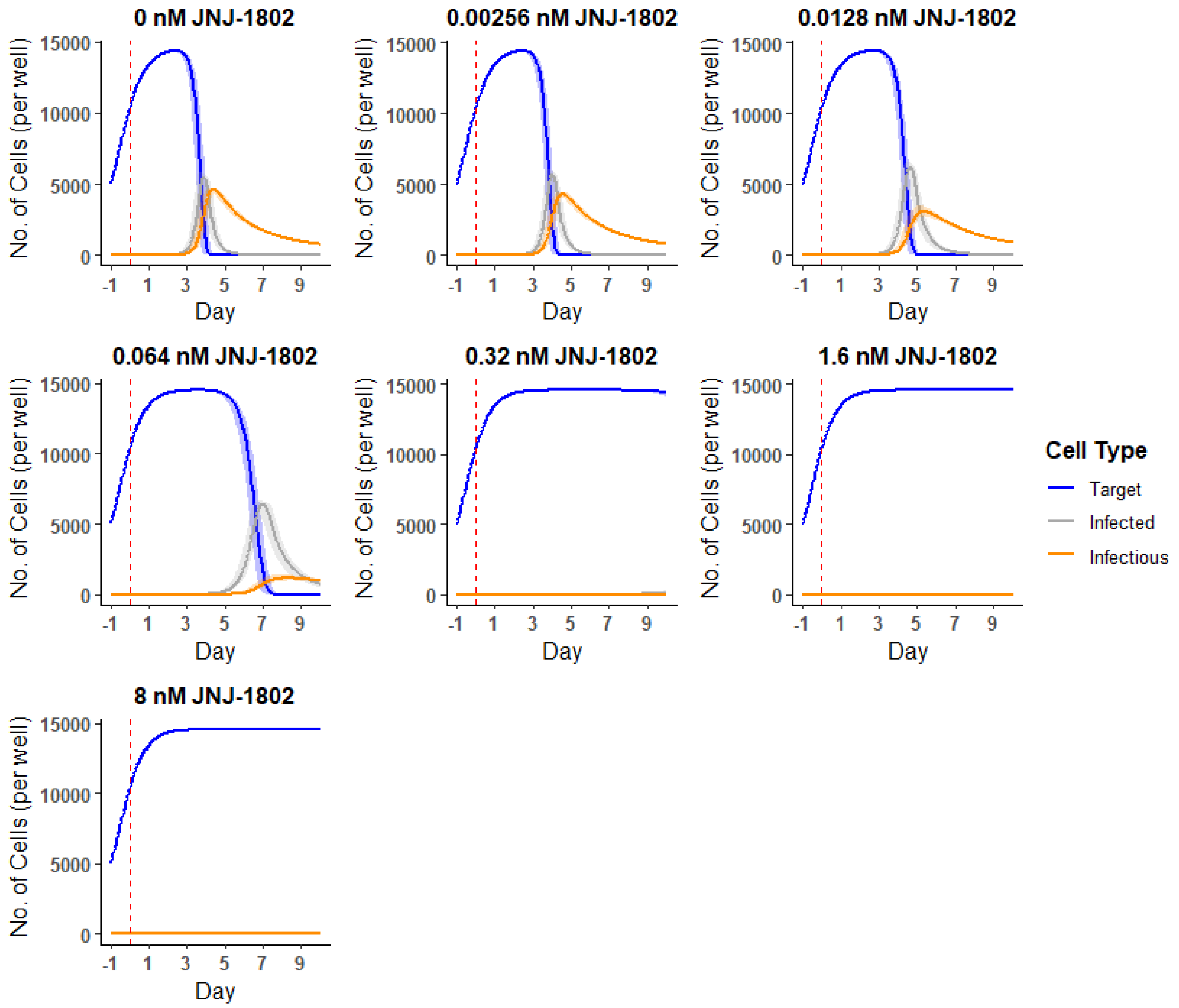

Fig E: Cell Dynamics (DENV-2/16681 strain, no refresh). Underlying modelled cell dynamics for the DENV-2/16681 strain with no medium refresh and antiviral concentrations of 0 nM, $2.56\text{x}10^{-03}$ nM, $1.28\text{x}10^{-02}$ nM, $6.40\text{x}10^{-02}$ nM, 0.32 nM, 1.6 nM, and 8 nM. The vertical red line indicates the time the viral inoculum was added to each well. The modelled dynamics of the target cells are in blue, those of the infected cells are in grey and those of the infectious (virion producing) cells are in orange; solid lines represent the median and the shading represents the 95% CrI. We assumed that the antiviral directly inhibits the transition process of infected cells to infectious (virion producing) cells , i.e., acts on τ .

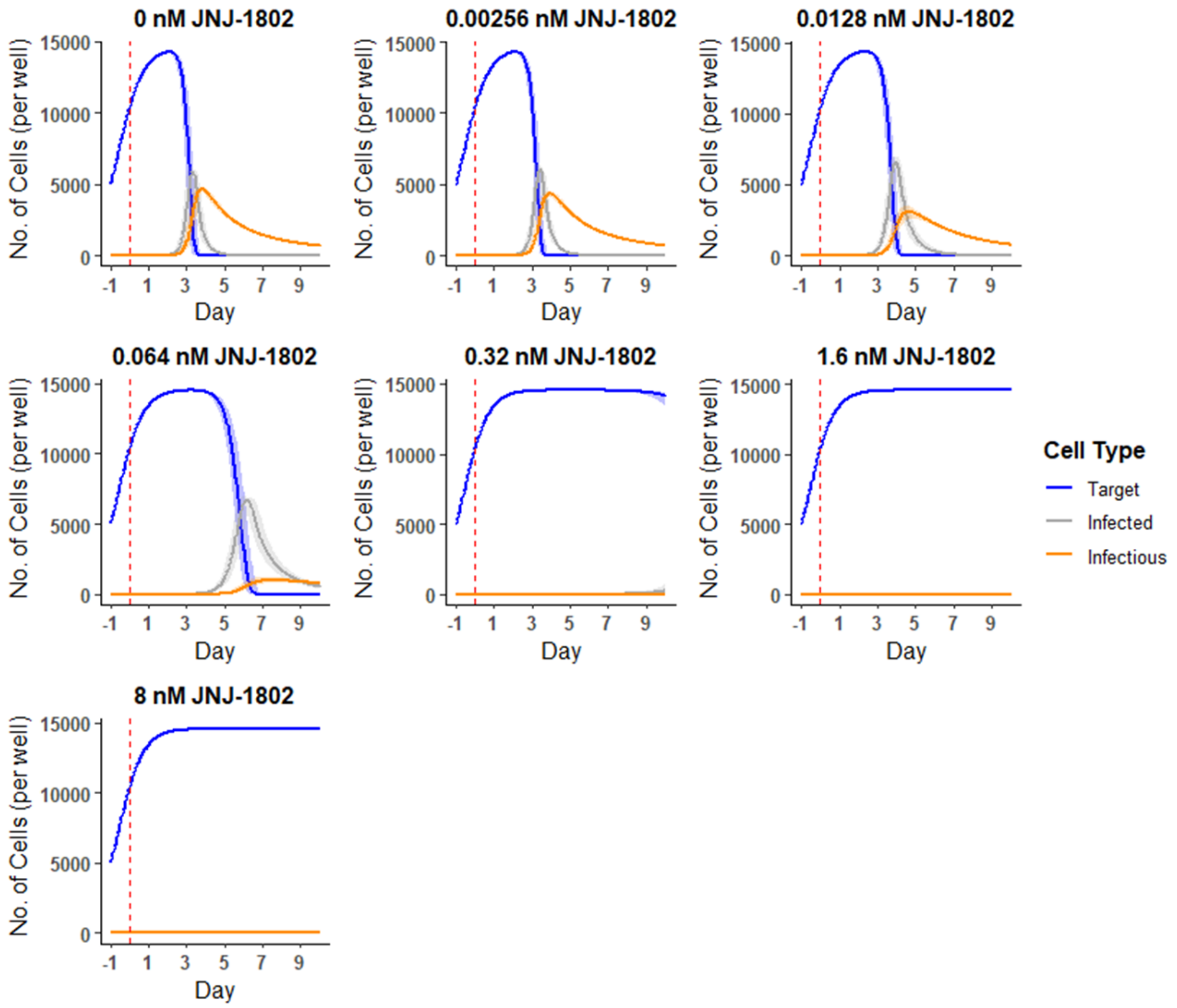

Fig F: Model Fits (DENV-2/RL strain, no refresh). Underlying modelled cell dynamics for the DENV-2/RL strain with no medium refresh and antiviral concentrations of 0 nM, 2.56×10^{-03} nM, 1.28×10^{-02} nM, 6.40×10^{-02} nM 0.32 nM, 1.6 nM, and 8 nM. The vertical red line indicates the time the viral inoculum was added to each well. The modelled dynamics of the target cells are in blue, those of the infected cells are in grey and those of the infectious (virion producing) cells are in orange; solid lines represent the median and the shading represents the 95% CrI. We assumed that the antiviral directly inhibits the transition process of infected cells to infectious (virion producing) cells , i.e., acts on τ .

2.3 Goodness of Fit

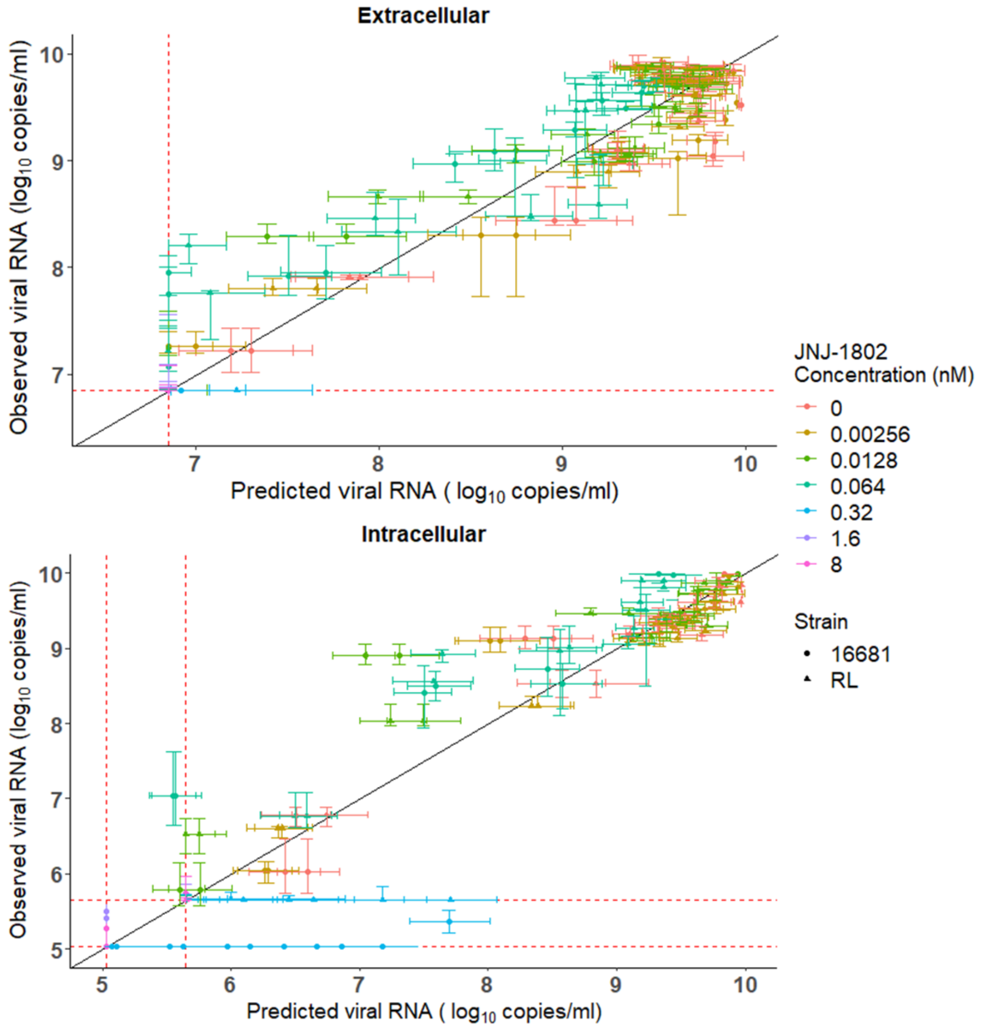

Fig G: Goodness of Fit. Observed vs predicted extracellular and intracellular RNA values for the experimental infection studies conducted, disaggregated by JNJ-1802 concentration and DENV-2 strain. Here, intracellular measurements below the limit of quantification (LOQ) were included during model fitting and measurements below LOD were left-censored at the LOD during model fitting. We assumed that the antiviral directly inhibits the transition process of infected cells to infectious (virion producing) cells i.e., acts on τ. For the observed values, we plot the median observed value across individual wells and the corresponding 2.5-97.5 percentiles. For the predicted values, we plot the median predicted value and corresponding 95% credible interval. For both observed and predicted values, we plot values below the LOD at the LOD (dashed red horizontal and vertical lines) to aid visual comparison between the observed and predicted values.

2.4 Limit of Quantification

		DENV-2/RL (No Refresh)		DENV-2/16681 (No Refresh)	
**Parameter**	**Description**	**Left-censoring at LOD**	**Left-censoring at LOQ**	**Left-censoring at LOD**	**Left-censoring at LOQ**
**β**	Infection rate of target cells per virion (day^{-1})	$3.18x10^{-08}$ ($2.29x10^{-08}$,$4.40x10^{-08}$)	$3.36x10^{-08}$ ($2.77x10^{-08}$,$4.25x10^{-08}$)	$1.32x10^{-08}$ ($9.05x10^{-09}$,$1.88x10^{-08}$)	$1.28x10^{-08}$ ($1.13x10^{-08}$,$1.52x10^{-08}$)
**ω**	Intracellular virus production rate per infectious cell (day^{-1})	$3.17x10^{6}$ ($2.32x10^{6}$,$4.44x10^{6}$)	$2.93x10^{+06}$ ($2.47x10^{+06}$,$3.44x10^{+06}$)	$1.36x10^{7}$ ($9.43x10^{6}$,$1.89x10^{7}$)	$1.65x10^{+07}$ ($1.37x10^{+07}$,$2.04x10^{+07}$)
**p**	Proportion of intracellular RNA becoming extracellular virus	$5.90x10^{-01}$ ($3.99x10^{-01}$,$8.83x10^{-01}$)	$5.12x10^{-01}$ ($3.54x10^{-01}$,$7.27x10^{-01}$)	$2.28x10^{-01}$ ($1.55x10^{-01}$,$3.55x10^{-01}$)	$1.93x10^{-01}$ ($1.69x10^{-01}$,$2.36x10^{-01}$)
**IC_{50}**	Concentration at which 50% of maximum effect is achieved (nM)	$1.23x10^{-02}$ ($8.33x10^{-03}$,$1.66x10^{-02}$)	$2.35x10^{-02}$ ($1.91x10^{-02}$,$2.91x10^{-02}$)	$1.28x10^{-02}$ ($9.00x10^{-03}$,$1.79x10^{-02}$)	$2.74x10^{-02}$ ($2.55x10^{-02}$,$2.93x10^{-02}$)
**h**	Hill coefficient	1.28 (1.14,1.42)	1.63 (1.47,1.88)	1.17 (1.07,1.31)	1.82 (1.73,1.94)
**R_0**	Basic reproduction number	277.01 (231.78,332.66)	235.32 (207.39,269.18)	191.10 (161.84,232.76)	195.30 (177.14,214.20)
**σ_V**	Residual error standard deviation (extracellular measurements)	0.83 (0.78,0.88)	0.90 (0.84,0.95)	0.83 (0.78,0.88)	0.90 (0.84,0.95)
**σ_X**	Residual error standard deviation (intracellular measurements)	0.91 (0.86,0.97)	0.40 (0.38,0.43)	0.91 (0.86,0.97)	0.40 (0.38,0.43)
**L**	Log-likelihood	-1619.87 (-1627.66,-1614.25)	-1046.64 (-1053.14,-1040.79)	-1619.87 (-1627.66,-1614.25)	-1046.64 (-1053.14,-1040.79)
**DIC**	Deviance Information Criterion	3,254	2,110	3,254	2,110

Table B: Posterior Parameter Estimates. Posterior parameter estimates for models assuming left-censoring of the viral RNA data at the limit of detection or at the limit of quantification. The median posterior estimate is reported with the 95% credible interval (CrI) in brackets. Here, we assume the antiviral directly inhibits the transition process of infected cells to infectious (virion producing) cells, i.e., acts on τ .

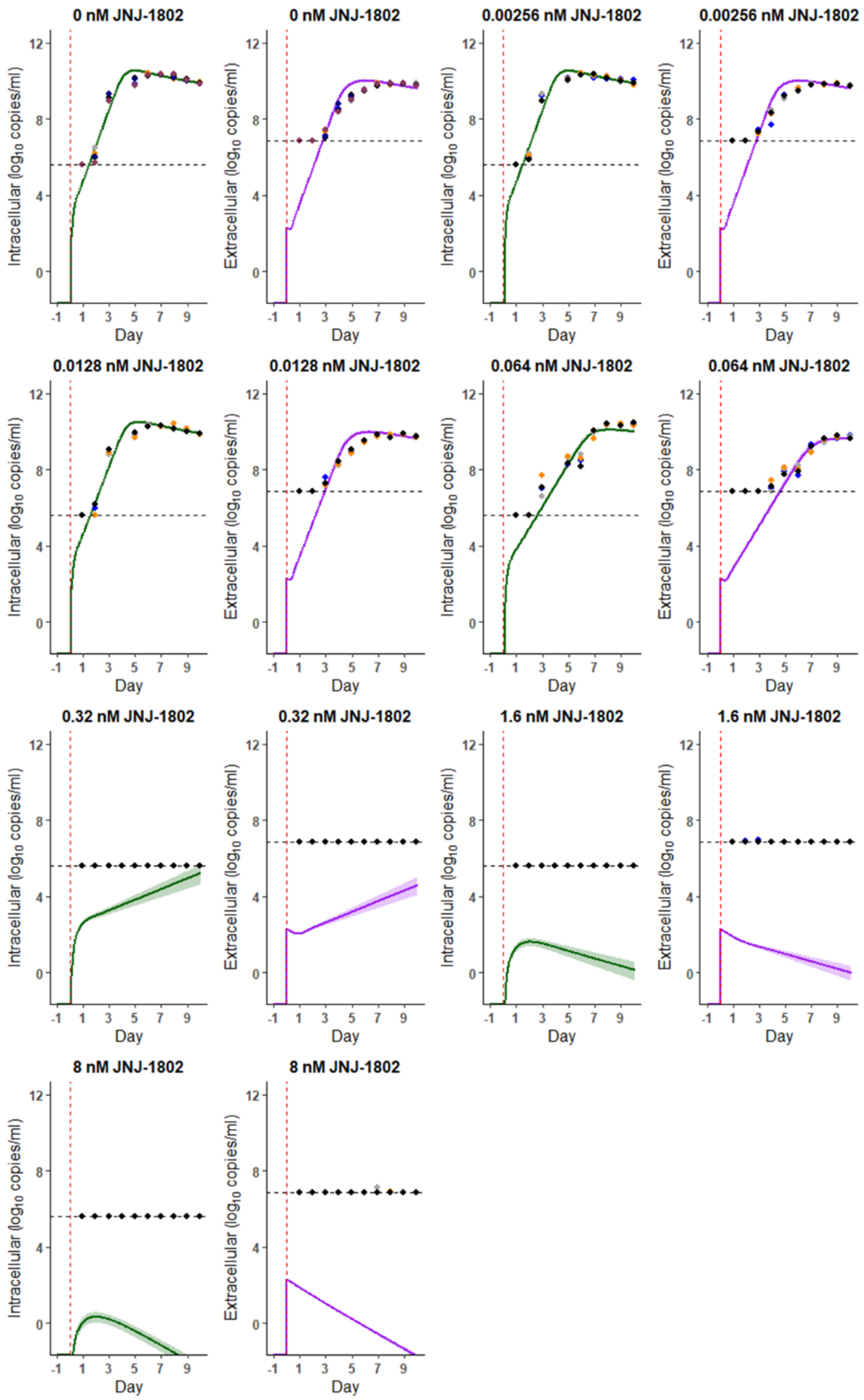

Fig H: Model Fits (DENV-2/16681 strain, no refresh). Model fits for the measurements observed using the DENV-2/16681 strain with no medium refresh and antiviral concentrations of 0 nM, 2.56x10^{-03} nM, 1.28x10^{-02} nM, 6.40x10^{-02} nM, 0.32 nM, 1.6 nM, and 8 nM. Coloured points represent the data from each well (6 wells for 0 nM, 4 wells for concentrations >0 nM), the vertical red line indicates the time the viral inoculum was added to each well and the horizontal dashed black line indicates the limit of quantification. The modelled dynamics of the intracellular RNA virus are in green and those of the extracellular RNA virus are in purple; solid lines represent the median and the shading represents the 95% CrI. Viral suppression is observed for concentrations of 1.6 nM and 8 nM. Here, measurements were left-censored at the LOQ during model fitting and we plot measurements below the LOQ (crosses in Figure 1) at the LOQ for visual display. We assumed that the antiviral inhibits the transition process of infected cells to infectious (virion producing) cells , i.e. acts on τ.

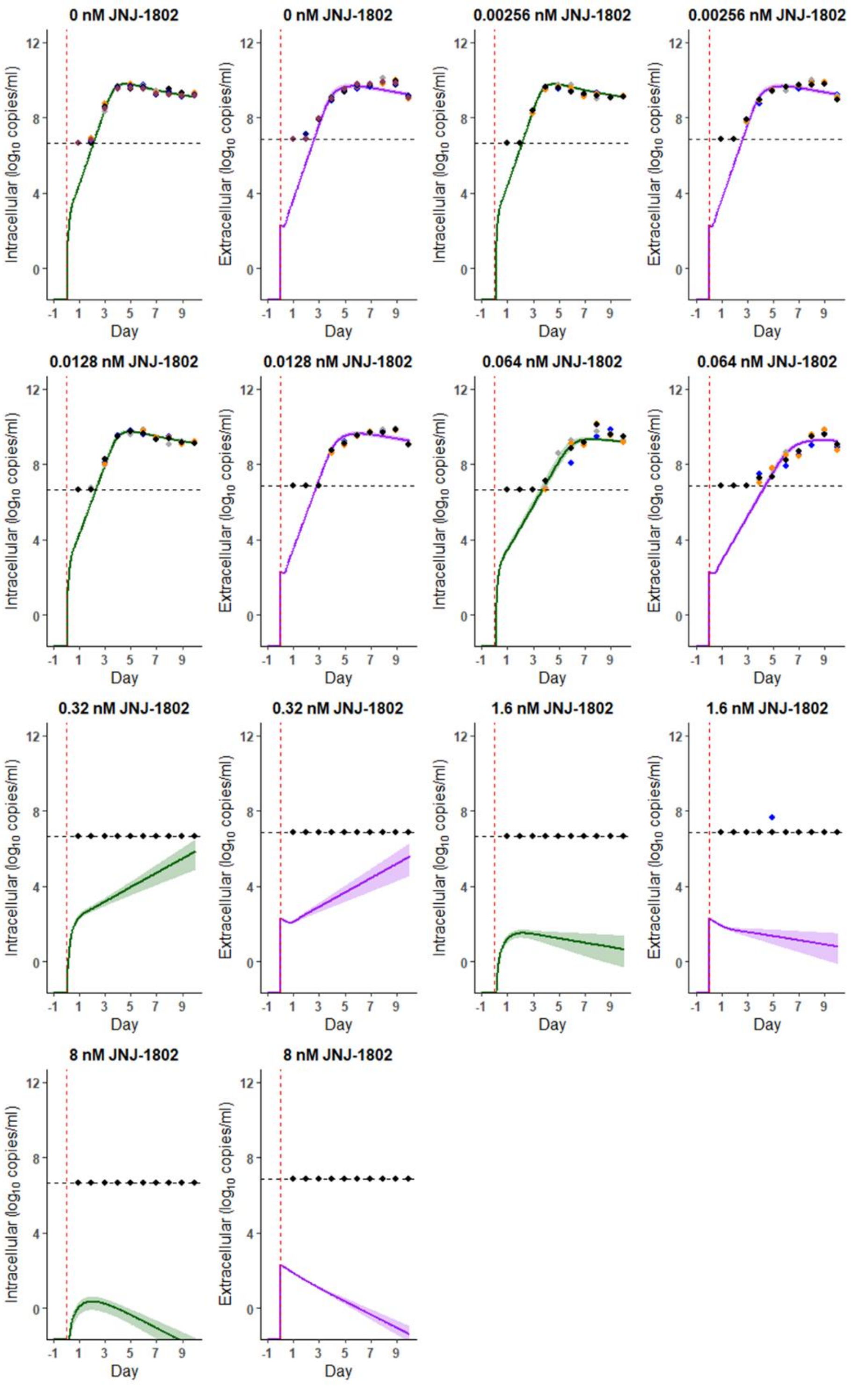

Fig I: Model Fits (DENV-2/RL strain, no refresh). Model fits for the measurements observed using the DENV-2/RL strain with no medium refresh and antiviral concentrations of 0 nM, 2.56×10^{-03} nM, 1.28×10^{-02} nM, 6.40×10^{-02} nM, 0.32 nM, 1.6 nM, and 8 nM. Coloured points represent the data from each well (6 wells for 0 nM, 4 wells for concentrations >0 nM), the vertical red line indicates the time the viral inoculum was added to each well and the horizontal dashed black line indicates the limit of quantification. The modelled dynamics of the intracellular RNA virus are in green and those of the extracellular RNA virus are in purple; solid lines represent the median and the shading represents the 95% CrI. Viral suppression is observed for concentrations of 1.6 nM and 8 nM. Here, measurements were left-censored at the LOQ during model fitting and we plot measurements below the LOQ (crosses in Figure 1) at the LOQ for visual display. We assumed that the antiviral inhibits the transition process of infected cells to infectious (virion producing) cells , i.e. acts on τ.

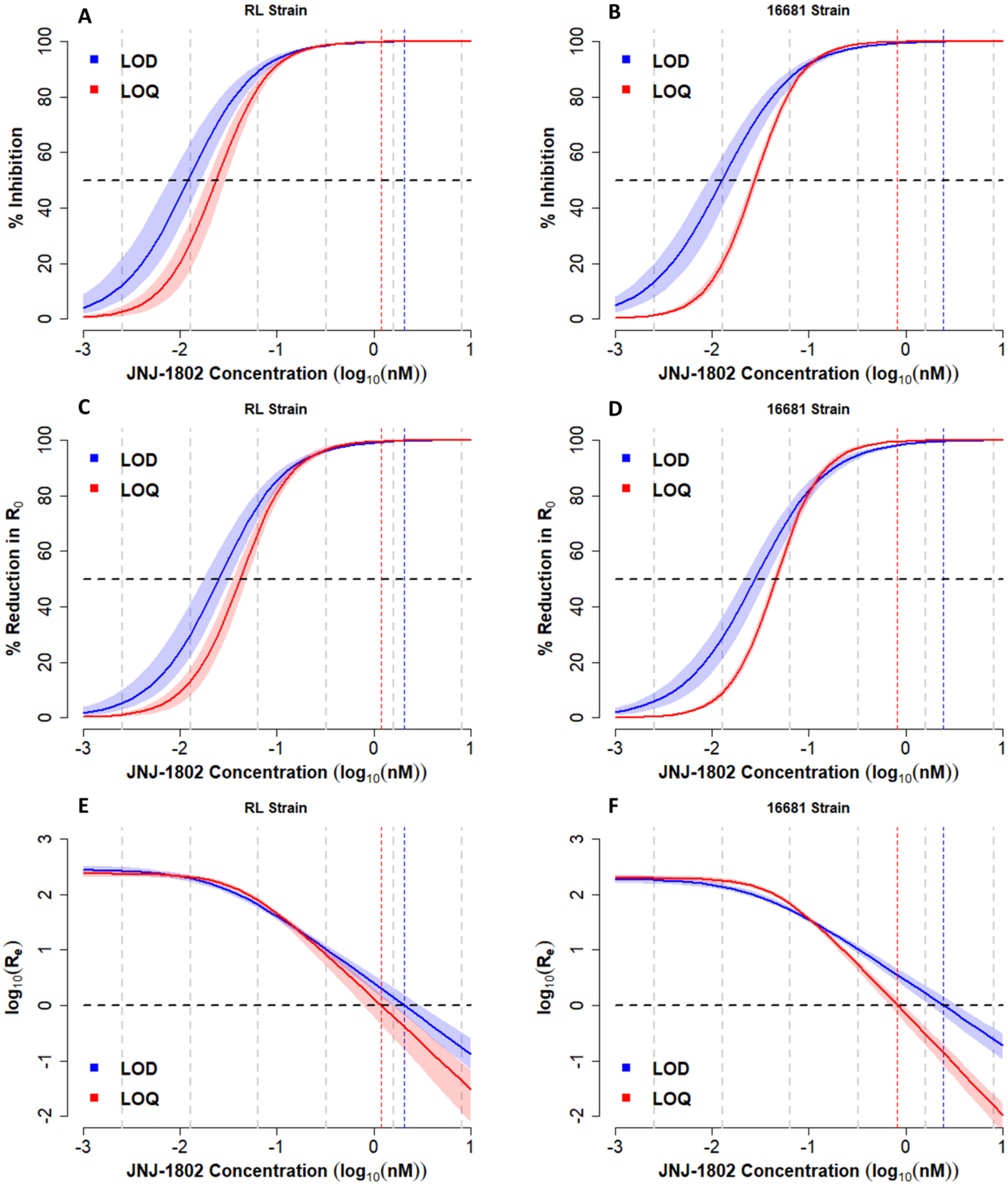

Fig J: Limit of Quantification (No Refresh). Estimated inhibition percentage (A,B), percentage reduction in the basic reproduction number R_0 (C,D) and effective reproduction number R_e (E,F) as a function of concentration for the DENV-2/RL strain (A,C,E) and DENV-2/16681 strain (B,D,F) where measurements were left-censored at either the limit of detection (LOD) or the limit of quantification (LOQ) and the well medium was not refreshed. Estimates were calculated by substituting 1,000 parameter values sampled from the posterior distribution into equations (1), (6) and (7) in the main text. Solid lines represent the median, the shading represents the 95% CrI, dotted grey vertical lines indicate the concentrations tested in the in vitro experiments, and the dotted blue and red vertical lines indicate the median concentration such the R_e =1. The dotted black horizontal lines indicate the threshold for a 50% reduction (A,B,C,D), and when R_e =1 (E,F). Here, we assume the antiviral directly inhibits the transition process of infected cells to infectious (virion producing) cells, i.e. acts on τ.

3 Alternative Mode of Drug Action

3.1 Mathematical Model

The flow diagram of the model provided in Figure K below describes the *in-vitro* dynamics of dengue virus following viral inoculation in each well assuming the antiviral directly inhibits viral production at the intracellular level (ω) rather than acting on the transition process of infected cells to infectious (virion producing) cells (τ) , as assumed in the main model. Table 3 in the main text provides a summary of the parameters used in the model.

We assume that target cells T replicate at a rate s_N and have a mean lifespan of $1/k_T$ days. However, as resources in individual wells were limited, we assume there is a limit to the size of the cell population each well can accommodate, and that regulation of the cell population is achieved via density-dependent mortality whereby mortality increases as the total number of cells reaches the carrying capacity (K_w) of the well.

Target cells become infected at a rate βV proportional to the abundance of V, the extracellular viral RNA concentration in the well (copies/mL). Following a latent period with a mean duration of $1/\tau$ days, infectious cells I synthesize intracellular viral RNA (X) at a net production rate ω (in absence of antiviral drug), a proportion *p* of which then gets secreted as extracellular viral RNA at a rate ε. Finally, extracellular RNA is assumed to decay at a rate k_V. We assume that infection increases the decay rate of cells by a fixed value, k_I.

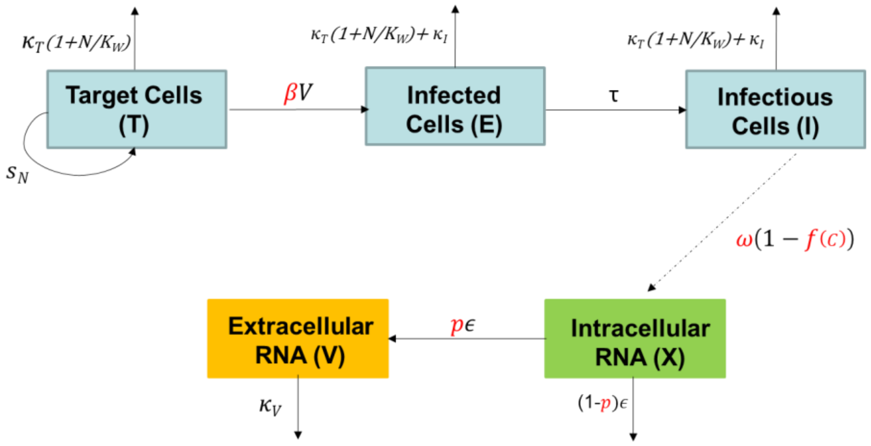

Fig K: Model Schematic. Target cells are infected at a rate β per virion. Following a latent period of 1/τ days on average, infectious cells I synthesize intracellular viral RNA (X) at a net production rate ω (in absence of antiviral drug), a proportion p of which then gets secreted as extracellular viral RNA at a rate ε. Extracellular viral RNA decays at a rate κ_V. Target cells replicate at a rate s_N; target cells and infected cells have a mean lifespan of $1/\kappa_T$ and $1/(\kappa_T+\kappa_I)$ days, respectively. Individual wells have a carrying capacity

K_W. The antiviral molecule acts on intracellular RNA production, following a concentration-dependent function f(C). The parameters in red are estimated, those in black are fixed.

We assume that the antiviral directly inhibits the production of intracellular RNA (ω). As the drug concentration remained constant throughout the course of each experiment, we assume that the magnitude of the effect f(C) also remained constant throughout the course of each experiment. We capture the relationship between the antiviral drug concentration C (nM) and the magnitude of the effect $f(C)$ using a Hill function[62,63] as follows:

$$f(C) = \frac{I_{max}\, C^h}{(IC_{50})^h + C^h} \in [0,1] \tag{1}$$

where $I_{max} \in [0,1]$ denotes the maximum inhibitory efficacy of the antiviral, IC_{50} denotes the concentration which achieves 50% of the maximum inhibitory effect and h denotes the Hill coefficient.

The dynamics of the cell population in each well are described by the following set of deterministic equations:

$$\begin{aligned}
\frac{dT}{dt} &= s_N\, T - k_T \left(1 + \frac{N}{K_w}\right) T - \beta V\, T \\
\frac{dE}{dt} &= \beta V\, T - k_T \left(1 + \frac{N}{K_w}\right) E - k_I E - \tau\ E \\
\frac{dI}{dt} &= \tau\, E - k_T \left(1 + \frac{N}{K_w}\right) I - k_I I \\
\frac{dX}{dt} &= \omega\big(1 - f(C)\big)\ I - \epsilon\, X \\
\frac{dV}{dt} &= p\epsilon\, X - k_V\, V
\end{aligned} \tag{2}$$

where $N = T + E + I$. The carrying capacity of the well K_w is obtained by considering the equilibrium cell population N^* in each well under disease free conditions (E=I=V=0). For an equilibrium population N^* we have that $s_N - k_T\ (1 + N^*/K_w) = 0$, and hence

$$K_w = \frac{k_T\, N^*}{s_N - k_T} \tag{3}$$

The basic reproduction number ($\mathcal{R}_0$) for this model is defined as the mean number of infected cells produced by each infected cell over its lifespan at under disease free conditions (at the start of infection, time t*) and without therapeutic intervention and is given by:

$$\mathcal{R}_0 = \frac{\beta \mathrm{T}^* \tau \omega p}{k_I^*(k_I^* + \tau) k_V} \tag{4}$$

where $k_I^* = k_T\left(1 + \frac{\mathrm{T}^*}{K_w}\right) + k_I$ and

$$\mathrm{T}^* = \mathrm{T}(\mathrm{t}^*) = \frac{K_w(s_N - k_T)T_0}{k_T T_0 + [K_w(s_N - k_T) - k_T T_0]e^{(-(s_N - k_T)t^*)}} \qquad (5)$$

denotes the target cell population at the start of infection (time t*). A full derivation of $\mathcal{R}_0$ is provided in Section 6 below.

We define the effective reproduction number ($\mathcal{R}_e$) as follows:

$$\mathcal{R}_e = \frac{\beta \mathrm{T}^* \tau \omega (1 - f(C))p}{k_I^*(k_I^* + \tau)k_V} \qquad (6)$$

where f(C) is defined as in equation (1) above.

For a given concentration C, we estimate the percentage reduction in the basic reproduction number $e(C)$ as follows

$$e(C) = 1 - \frac{\mathcal{R}_e}{\mathcal{R}_0} = f(C) \qquad (7)$$

Using equations (1) and (6) above, we have that a concentration C_{crit} with

$$C_{crit} \geq \left(\frac{z_1}{I_{max} z_2 - z_1}\right)^{1/h} IC_{50} \qquad (8)$$

where

$$z_1 = \omega p\, \beta\, \mathrm{T}^* \tau - k_I^*\, k_V (k_I^* + \tau),\ z_2 = \omega p\, \beta\, \mathrm{T}^* \tau$$

is required to bring the effective reproduction number ($\mathcal{R}_e$) below 1.

For the model without a latent stage, we have that

$$\mathcal{R}_e = \frac{\beta \mathrm{T}^* \tau \omega (1 - f(C))p}{k_I^* k_V} \qquad (9)$$

and a concentration C_{crit} with

$$C_{crit} \geq \left(\frac{z_1}{I_{max} z_2 - z_1}\right)^{1/h} IC_{50} \qquad (10)$$

where

$$z_1 = \omega p\, \beta\, \mathrm{T}^* - k_I^*\, k_V, z_2 = \omega p\, \beta\, \mathrm{T}^*$$

is required to bring the effective reproduction number ($\mathcal{R}_e$) below 1.

3.2 Model Fits

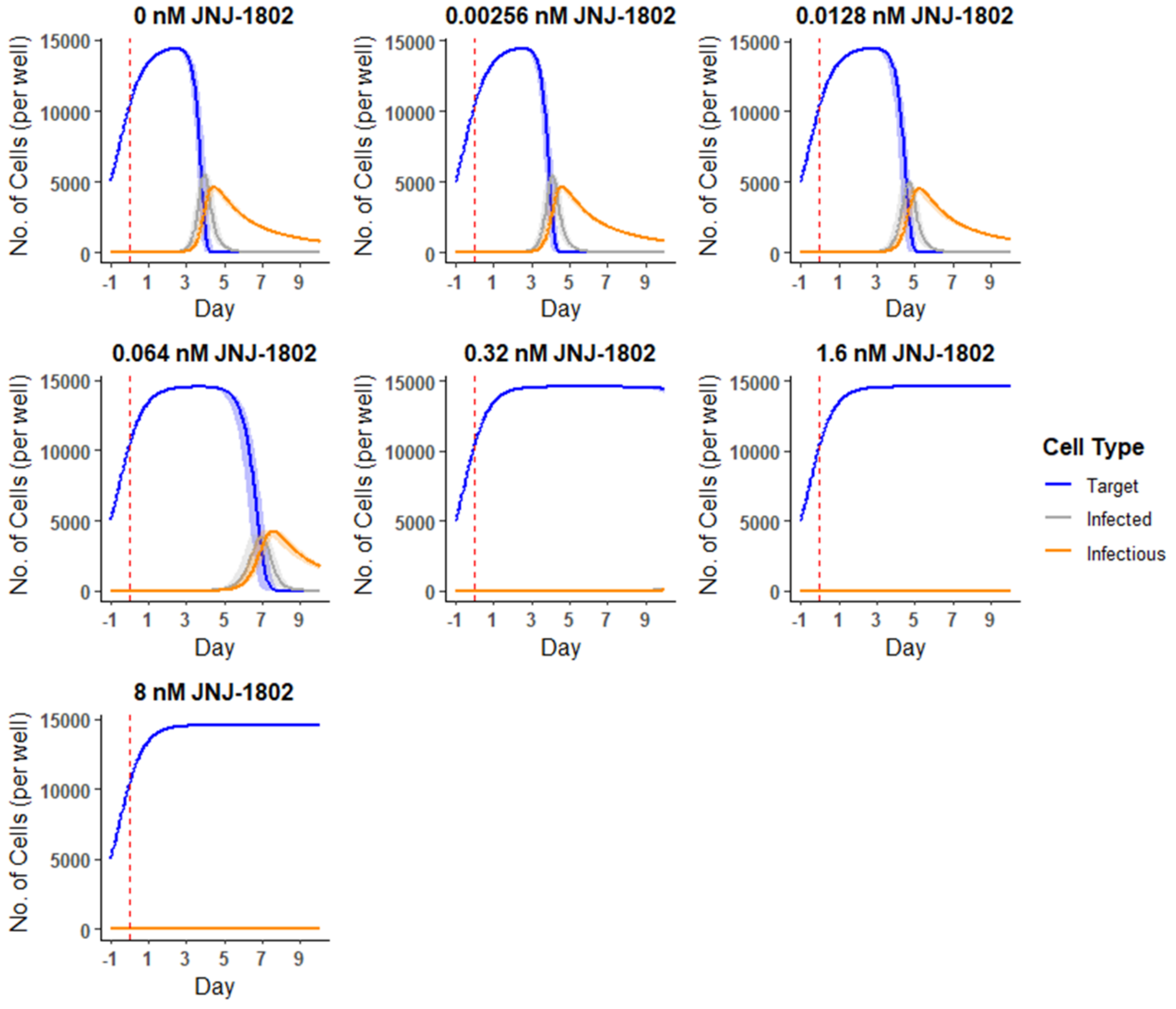

Fig L: Cell Dynamics (DENV-2/16681 strain, no refresh). Underlying modelled cell dynamics for the DENV-2/16681 strain with no medium refresh and antiviral concentrations of 0 nM, $2.56x10^{-03}$ nM, $1.28x10^{-02}$ nM, $6.40x10^{-02}$ nM, 0.32 nM, 1.6 nM, and 8 nM. The vertical red line indicates the time the viral inoculum was added to each well. The modelled dynamics of the target cells are in blue, those of the infected cells are in grey and those of the infectious (virion producing) cells are in orange; solid lines represent the median and the shading represents the 95% CrI. We assumed that the antiviral directly inhibits intracellular RNA production, i.e., acts on ω.

.

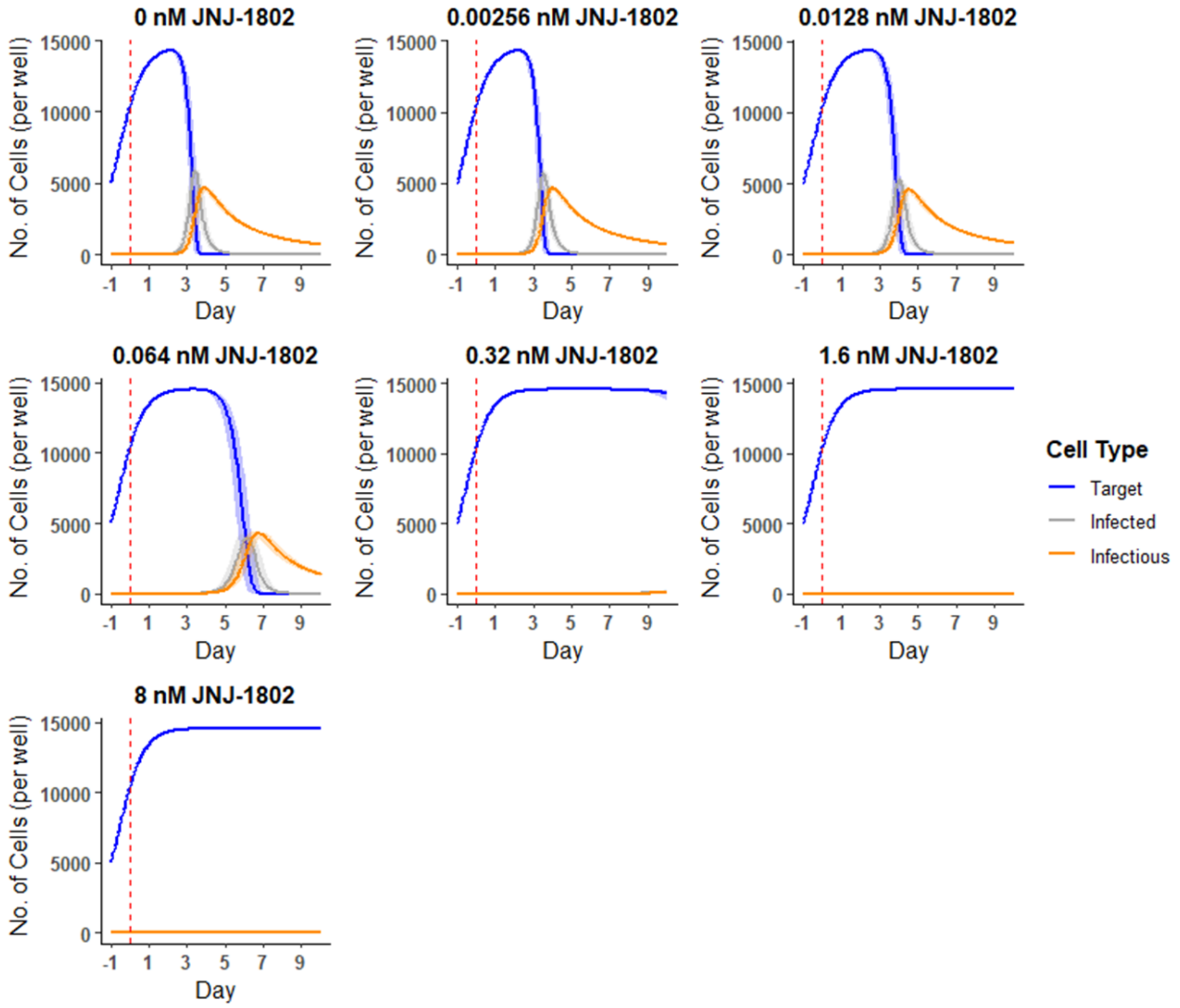

Fig M: Cell Dynamics (DENV-2/RL strain, no refresh). Underlying modelled cell dynamics for the DENV-2/16681 strain with no medium refresh and antiviral concentrations of 0 nM, 2.56×10^{-03} nM, 1.28×10^{-02} nM, 6.40×10^{-02} nM, 0.32 nM, 1.6 nM, and 8 nM. The vertical red line indicates the time the viral inoculum was added to each well. The modelled dynamics of the target cells are in blue, those of the infected cells are in grey and those of the infectious (virion producing) cells are in orange; solid lines represent the median and the shading represents the 95% CrI. We assumed that the antiviral directly inhibits intracellular RNA production, i.e., acts on ω.

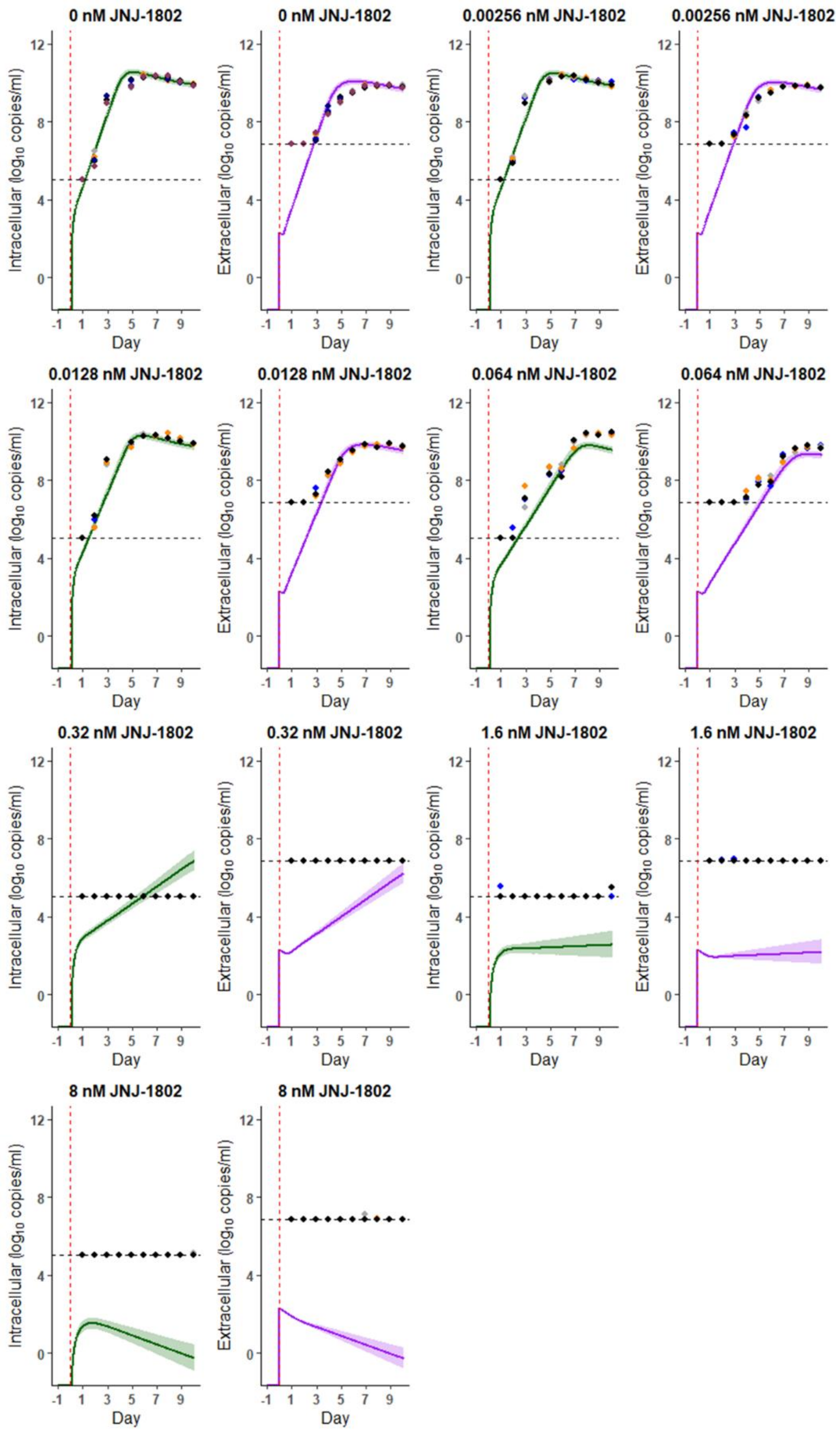

Fig N: Model Fits (DENV-2/16681 strain, no refresh). Model fits for the measurements observed using the DENV-2/16681 strain with no medium refresh and antiviral concentrations of 0 nM, 2.56x10^{-03} nM, 1.28x10^{-02} nM, 6.40x10^{-02} nM 0.32 nM, 1.6 nM, and 8 nM. Coloured points represent the data from each well (6 wells for 0 nM, 4 wells for concentrations >0 nM), the vertical red line indicates the time the viral inoculum was added to each well and the horizontal dashed black line indicates the limit of detection. The modelled dynamics of the intracellular RNA virus are in green and those of the extracellular RNA virus are in purple; solid lines represent the median and the shading represents the 95% CrI. Here, measurements below the limit of quantification (crosses in Figure 1) were included during model fitting. Measurements below the LOD were left censored at the LOD during model fitting and we plot these measurements at the LOD for visual display. We assumed that the antiviral directly inhibits intracellular RNA production, i.e., acts on ω.

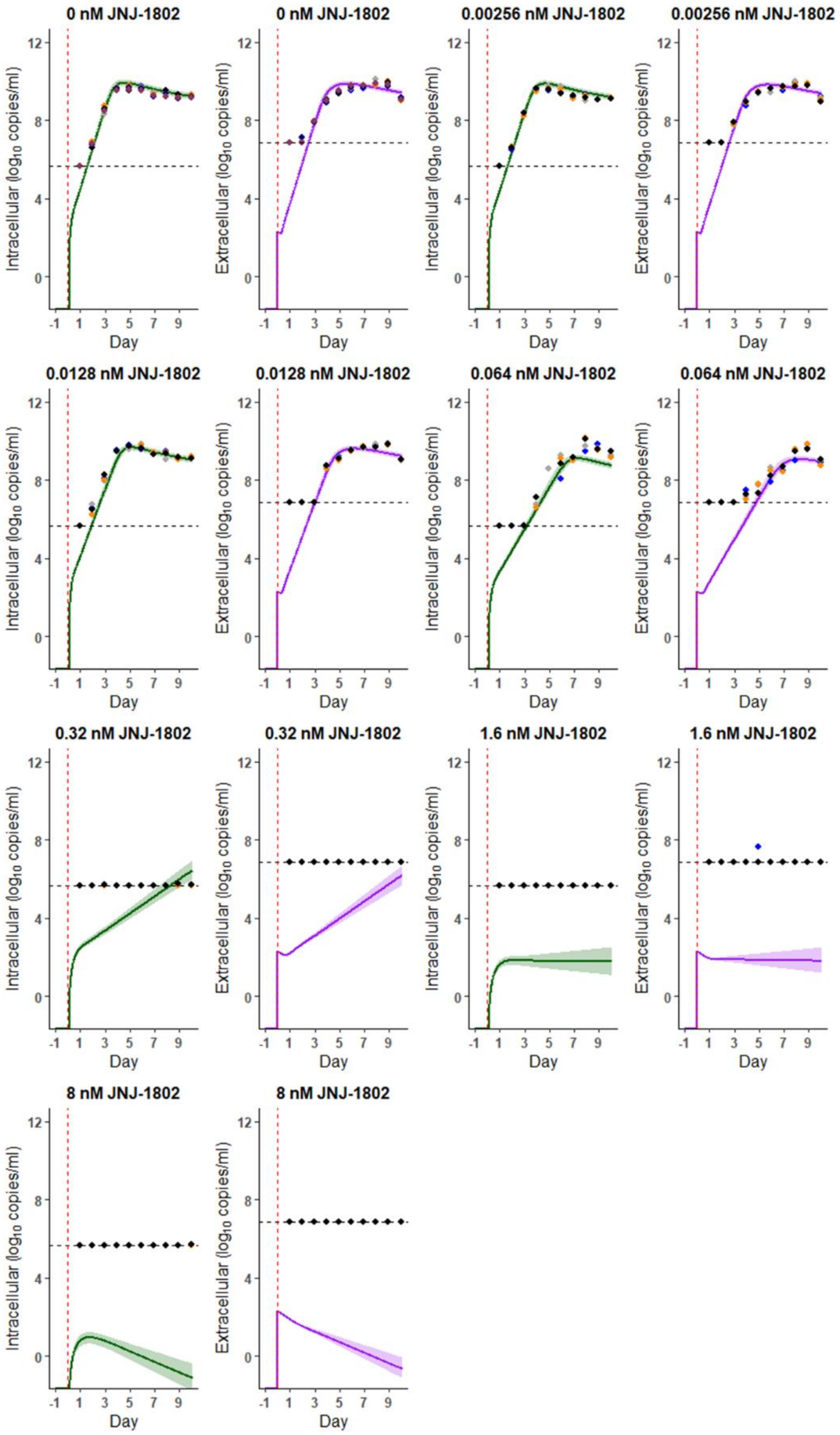

Fig O: Model Fits (DENV-2/RL strain, no refresh). Model fits for the measurements observed using the DENV-2/RL strain with no medium refresh and antiviral concentrations of 0 nM, 2.56×10^{-03} nM, 1.28×10^{-02} nM, 6.40×10^{-02} nM, 0.32 nM, 1.6 nM, and 8 nM. Coloured points represent the data from each well (6 wells for 0 nM, 4 wells for concentrations >0 nM), the vertical red line indicates the time the viral inoculum was added to each well and the horizontal dashed black line indicates the limit of detection. The modelled dynamics of the intracellular RNA virus are in green and those of the extracellular RNA virus are in purple; solid lines represent the median and the shading represents the 95% CrI. Here, measurements below the limit of quantification (crosses in Figure 1) were included during model fitting. Measurements below the LOD were left censored at the LOD during model fitting and we plot these measurements at the LOD for visual display. We assumed that the antiviral directly inhibits intracellular RNA production, i.e., acts on ω.

Parameter	Description	RL (No Refresh)			16681 (No Refresh)		
		Action on τ	Action on ω (no latent period)	Action on ω (with latent period)	Action on τ	Action on ω (no latent period)	Action on ω (with latent period)
**β**	Infection rate of target cells per virion (day^{-1})	3.18×10^{-08} (2.29×10^{-08},4.40×10^{-08})	7.04×10^{-09} (5.11×10^{-09},9.46×10^{-09})	2.39×10^{-08} (1.74×10^{-08},3.29×10^{-08})	1.32×10^{-08} (9.05×10^{-09},1.88×10^{-08})	3.32×10^{-09} (2.50×10^{-09},4.62×10^{-09})	1.05×10^{-08} (7.83×10^{-09},1.57×10^{-08})
**ω**	Intracellular virus production rate per infectious cell (day^{-1})	3.17×10^{6} (2.32×10^{6},4.44×10^{6})	3.00×10^{6} (2.15×10^{6},4.56×10^{6})	4.04×10^{6} (2.79×10^{6},5.79×10^{6})	1.36×10^{7} (9.43×10^{6},1.89×10^{7})	1.28×10^{7} (9.25×10^{6},1.80×10^{7})	1.68×10^{7} (1.13×10^{7},2.42×10^{7})
**p**	Proportion of intracellular RNA becoming extracellular virus	5.90×10^{-01} (3.99×10^{-01},8.83×10^{-01})	5.44×10^{-01} (3.43×10^{-01},8.12×10^{-01})	5.75×10^{-01} (3.90×10^{-01},8.47×10^{-01})	2.28×10^{-01} (1.55×10^{-01},3.55×10^{-01})	2.10×10^{-01} (1.45×10^{-01},2.94×10^{-01})	2.21×10^{-01} (1.52×10^{-01},3.62×10^{-01})
**IC_{50}**	Concentration at which 50% of maximum effect is achieved (nM)	1.23×10^{-02} (8.33×10^{-03},1.66×10^{-02})	1.91×10^{-02} (1.26×10^{-02},2.80×10^{-02})	1.61×10^{-02} (1.05×10^{-02},2.27×10^{-02})	1.28×10^{-02} (9.00×10^{-03},1.79×10^{-02})	2.03×10^{-02} (1.44×10^{-02},2.74×10^{-02})	1.64×10^{-02} (1.19×10^{-02},2.28×10^{-02})
**h**	Hill coefficient	1.28 (1.14,1.42)	1.04 (0.91,1.18)	1.20 (1.06,1.35)	1.17 (1.07,1.31)	0.97 (0.86,1.09)	1.09 (0.97,1.22)
**R0**	Basic reproduction number	277.01 (231.78,332.66)	90.47 (77.21,105.89)	263.40 (219.73,316.49)	191.10 (161.84,232.76)	70.05 (61.94,80.07)	187.15 (158.18,221.16)
**σ_V**	Residual error standard deviation (extracellular measurements)	0.83 (0.78,0.88)	0.85 (0.80,0.90)	0.83 (0.78,0.89)	0.83 (0.78,0.88)	0.85 (0.80,0.90)	0.83 (0.78,0.89)
**σ_X**	Residual error standard deviation (intracellular measurements)	0.91 (0.86,0.97)	0.93 (0.88,0.98)	0.95 (0.89,1.01)	0.91 (0.86,0.97)	0.93 (0.88,0.98)	0.95 (0.89,1.01)
**L**	Log-likelihood	-1619.87 (-1627.66,-1614.25)	-1643.69 (-1651.00,-1638.63)	-1647.13 (-1654.86,-1642.37)	-1619.87 (-1627.66,-1614.25)	-1643.69 (-1651.00,-1638.63)	-1647.13 (-1654.86,-1642.37)
**DIC**	Deviance Information Criterion	3,254	3,298	3,306	3,254	3,298	3,306

Table C: Posterior Parameter Estimates. Posterior parameter estimates for different modes of drug action and model structure – (1) inhibition of transition process of infected cells to infectious (virion producing) cells (action on τ), (2) direct inhibition of intracellular RNA production in a model with no latent period allowing for the transition process of infected cells to infectious cells (action on ω, no latent period) and (3) direct inhibition of intracellular RNA production in a model with a latent period allowing for the transition process of infected cells to infectious cells (action on ω, with latent period) The median posterior estimate is reported with the 95% credible interval in brackets. Here, measurements below the limit of quantification but above the limit of detection (crosses in Figure 1) were included during model fitting.

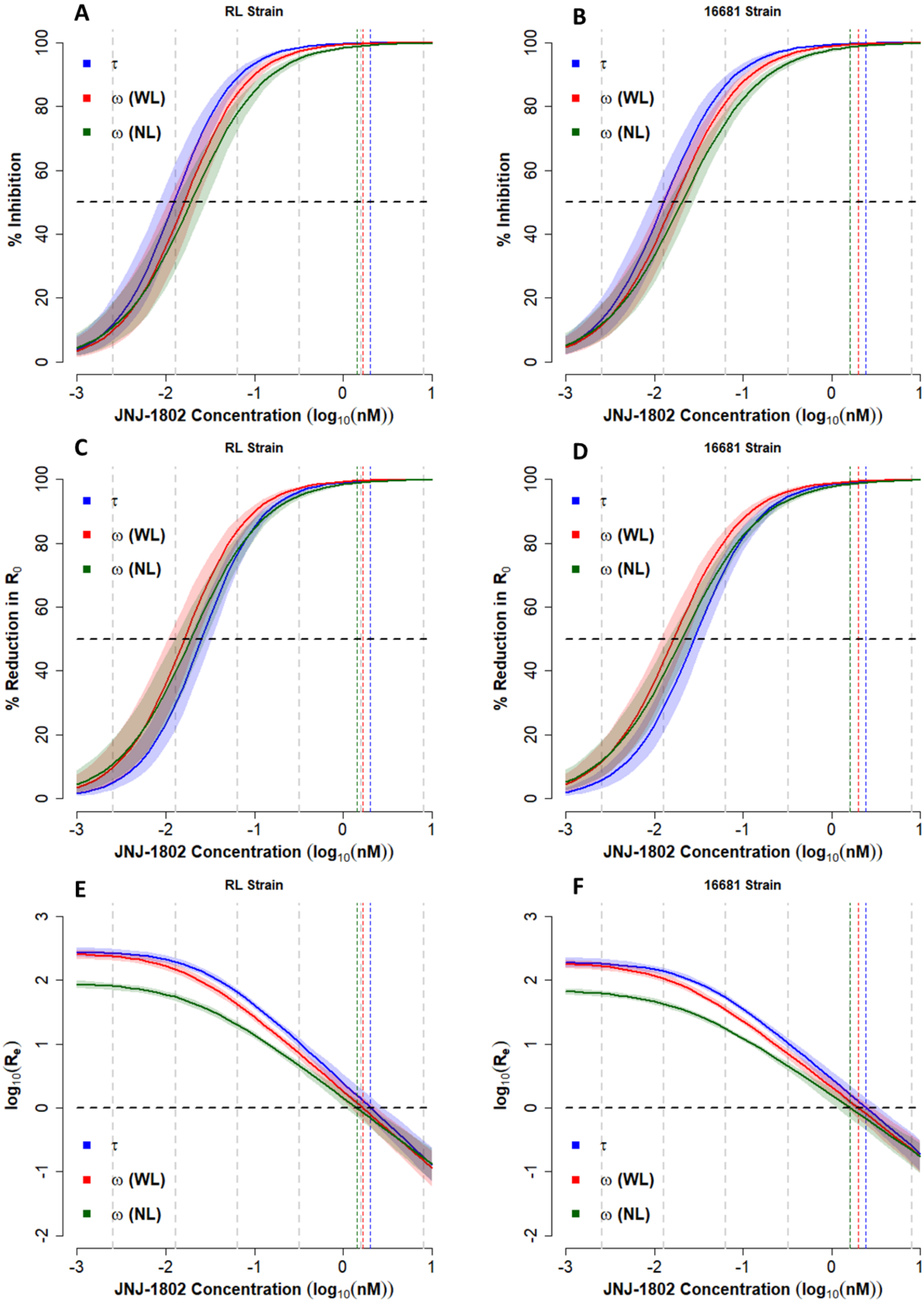

Fig P: Effect of antiviral (No Refresh). Estimated inhibition percentage (A,B), percentage reduction in the basic reproduction number R_0 (C,D) and effective reproduction number R_e (E,F) as a function of concentration, for the DENV-2/RL strain (A,C,E) and DENV-2/16681 strain (B,D,F) where the drug action was on intracellular RNA production in a model with/without a latent period (WL/NL) allowing for maturation of infected cells (action on ω) or the drug action was on the transition process of infected cells to infectious (virion producing) cells (action on τ) and the well medium was not refreshed. Estimates were calculated by substituting 1,000 parameter values sampled from the posterior distribution into equations (1), (6) and (7) in the main text and above. Solid lines represent the median, the shading represents the 95% CrI, dotted grey vertical lines indicate the concentrations tested in the in vitro experiments, and the dotted green, blue and red vertical lines indicate the median concentration such the R_e =1. The dotted black horizontal lines indicate the threshold for a 50% reduction (A,B,C,D), and when R_e =1 (E,F). Here, measurements below the limit of quantification (crosses in Figure 1) were included during model fitting.

4 Sensitivity to Viral Inoculum

		RL (No Refresh)			
**Parameter**	**Description**	**V_0=50**	**V_0=100**	**V_0=200**	**V_0=300**
**β**	Infection rate of target cells per virion (day^{-1})	$4.64x10^{-08}$ ($3.37x10^{-08}$,$7.15x10^{-08}$)	$3.77x10^{-08}$ ($2.65x10^{-08}$,$5.49x10^{-08}$)	$3.18x10^{-08}$ ($2.29x10^{-08}$,$4.40x10^{-08}$)	$2.80x10^{-08}$ ($1.94x10^{-08}$,$3.83x10^{-08}$)
**ω**	Intracellular virus production rate per infectious cell (day^{-1})	$3.33x10^{6}$ ($2.38x10^{6}$,$4.80x10^{6}$)	$3.42x10^{6}$ ($2.48x10^{6}$,$4.89x10^{6}$)	$3.17x10^{6}$ ($2.32x10^{6}$,$4.44x10^{6}$)	$3.17x10^{6}$ ($2.22x10^{6}$,$4.54x10^{6}$)
**p**	Proportion of intracellular RNA becoming extracellular virus	$5.38x10^{-01}$ ($2.76x10^{-01}$,$8.92x10^{-01}$)	$5.33x10^{-01}$ ($3.37x10^{-01}$,$8.50x10^{-01}$)	$5.90x10^{-01}$ ($3.99x10^{-01}$,$8.83x10^{-01}$)	$5.95x10^{-01}$ ($3.92x10^{-01}$,$8.79x10^{-01}$)
**IC_{50}**	Concentration at which 50% of maximum effect is achieved (nM)	$1.08x10^{-02}$ ($7.29x10^{-03}$,$1.66x10^{-02}$)	$1.18x10^{-02}$ ($7.77x10^{-03}$,$1.71x10^{-02}$)	$1.23x10^{-02}$ ($8.33x10^{-03,}1.66x10^{-02}$)	$1.24x10^{-02}$ ($8.32x10^{-03}$,$1.78x10^{-02}$)
**h**	Hill coefficient	1.27 (1.14,1.44)	1.28 (1.14,1.44)	1.28 (1.14,1.42)	1.27 (1.14,1.42)
**R0**	Basic reproduction number	399.12 (316.31,497.24)	324.36 (260.01,403.97)	277.01 (231.78,332.66)	246.34 (205.32,301.80)
**σ_V**	Residual error standard deviation (extracellular measurements)	0.90 (0.84,0.96)	0.86 (0.81,0.92)	0.83 (0.78,0.88)	0.80 (0.75,0.85)
**σ_X**	Residual error standard deviation (intracellular measurements)	0.96 (0.91,1.02)	0.93 (0.88,0.99)	0.91 (0.86,0.97)	0.90 (0.86,0.95)
**L**	Log-likelihood	-1683.49 (-1691.64,-1677.28)	-1650.91 (-1659.32,-1645.20)	-1619.87 (-1627.66,-1614.25)	-1602.35 (-1609.75,-1597.14)
**DIC**	Deviance Information Criterion	3,379	3,316	3,254	3,218

Table D: Posterior Parameter Estimates (RL Strain). Posterior parameter estimates for different starting values of the initial viral inoculum (V_0). Here, measurements below the limit of quantification (crosses in Figure 1) were included during model fitting. Measurements below the LOD were left censored at the LOD during model fitting and we assumed that the antiviral directly inhibits the transition process of infected cells to infectious (virion producing) cells , i.e., acts on τ.

		16681 (No Refresh)			
**Parameter**	**Description**	**V_0=50**	**V_0=100**	**V_0=200**	**V_0=300**
**β**	Infection rate of target cells per virion (day^{-1})	$4.64x10^{-08}$ ($3.37x10^{-08}$,$7.15x10^{-08}$)	$1.70x10^{-08}$ ($1.22x10^{-08}$,$2.41x10^{-08}$)	$1.32x10^{-08}$ ($9.05x10^{-09}$,$1.88x10^{-08}$)	$1.17x10^{-08}$ ($8.30x10^{-09}$,$1.67x10^{-08}$)
**ω**	Intracellular virus production rate per infectious cell (day^{-1})	$3.33x10^{6}$ ($2.38x10^{6}$,$4.80x10^{6}$)	$1.47x10^{7}$ ($1.02x10^{7}$,$2.11x10^{7}$)	$1.36x10^{7}$ ($9.43x10^{6}$,$1.89x10^{7}$)	$1.34x10^{7}$ ($9.00x10^{6}$,$1.92x10^{7}$)
**p**	Proportion of intracellular RNA becoming extracellular virus	$5.38x10^{-01}$ ($2.76x10^{-01}$,$8.92x10^{-01}$)	$1.97x10^{-01}$ ($1.34x10^{-01}$,$2.98x10^{-01}$)	$2.28x10^{-01}$ ($1.55x10^{-01}$,$3.55x10^{-01}$)	$2.40x10^{-01}$ ($1.50x10^{-01}$,$3.59x10^{-01}$)
**IC_{50}**	Concentration at which 50% of maximum effect is achieved (nM)	$1.08x10^{-02}$ ($7.29x10^{-03}$,$1.66x10^{-02}$)	$1.15x10^{-02}$ ($8.46x10^{-03}$,$1.60x10^{-02}$)	$1.28x10^{-02}$ ($9.00x10^{-03}$,$1.79x10^{-02}$)	$1.29x10^{-02}$ ($9.11x10^{-03}$,$1.73x10^{-02}$)
**h**	Hill coefficient	1.27 (1.14,1.44)	1.16 (1.05,1.30)	1.17 (1.07,1.31)	1.16 (1.04,1.29)
**R0**	Basic reproduction number	399.12 (316.31,497.24)	231.33 (192.32,276.56)	191.10 (161.84,232.76)	174.85 (146.28,205.00)
**$σ_V$**	Residual error standard deviation (extracellular measurements)	0.90 (0.84,0.96)	0.86 (0.81,0.92)	0.83 (0.78,0.88)	0.80 (0.75,0.85)
**$σ_X$**	Residual error standard deviation (intracellular measurements)	0.96 (0.91,1.02)	0.93 (0.88,0.99)	0.91 (0.86,0.97)	0.90 (0.86,0.95)
**L**	Log-likelihood	-1683.49 (-1691.64,-1677.28)	-1650.91 (-1659.32,-1645.20)	-1619.87 (-1627.66,-1614.25)	-1602.35 (-1609.75,-1597.14)
**DIC**	Deviance Information Criterion	3,379	3,316	3,254	3,218

Table E: Posterior Parameter Estimates (16681 Strain). Posterior parameter estimates for different starting values of the initial viral inoculum (V_0). Here, measurements below the limit of quantification (crosses in Figure 1) were included during model fitting. Measurements below the LOD were left censored at the LOD during model fitting and we assumed that the antiviral directly inhibits the transition process of infected cells to infectious (virion producing) cells , i.e., acts on τ.

5 Sensitivity to Data Excluded

Parameter	Description	RL (No Refresh)<br>Raw Data (no data excluded)	16681 Day 4 Included, outliers excluded	Outliers included, 16681 day 4 excluded	Final Dataset
**β**	Infection rate of target cells per virion (day^{-1})	2.96 x10^{-08} (2.06 x10^{-08},4.22 x10^{-08})	3.01 x10^{-08} (2.21 x10^{-08},4.44 x10^{-08})	2.99 x10^{-08} (2.01 x10^{-08},4.39 x10^{-08})	3.18x10^{-08} (2.29x10^{-08},4.40x10^{-08})
**ω**	Intracellular virus production rate per infectious cell (day^{-1})	3.14 x10^{6} (2.07 x10^{6},4.42 x10^{6})	3.32 x10^{6} (2.37 x10^{6},4.81 x10^{6})	3.19 x10^{6} (2.10 x10^{6},4.59 x10^{6})	3.17x10^{6} (2.32x10^{6},4.44x10^{6})
**p**	Proportion of intracellular RNA becoming extracellular virus	6.45 x10^{-01} (4.10 x10^{-01},9.36 x10^{-01})	5.75 x10^{-01} (3.89 x10^{-01},8.57 x10^{-01})	6.12 x10^{-01} (3.88 x10^{-01},9.10 x10^{-01})	5.90x10^{-01} (3.99x10^{-01},8.83x10^{-01})
**IC_{50}**	Concentration at which 50% of maximum effect is achieved (nM)	9.06 x10^{-03} (5.68 x10^{-03},1.33 x10^{-02})	1.24 x10^{-02} (6.87 x10^{-03},1.75 x10^{-02})	8.79 x10^{-03} (5.58 x10^{-03},1.51 x10^{-02})	1.23x10^{-02} (8.33x10$^{-03,}$1.66x10^{-02})
**h**	Hill coefficient	1.08 (0.96,1.21)	1.28 (1.10,1.45)	1.07 (0.95,1.24)	1.28 (1.14,1.42)
**R0**	Basic reproduction number	270.57 (225.55,330.09)	273.14 (227.57,335.63)	269.57 (219.44,328.39)	277.01 (231.78,332.66)
**σ_V**	Residual error standard deviation (extracellular measurements)	0.88 (0.82,0.93)	0.82 (0.77,0.87)	0.89 (0.83,0.94)	0.83 (0.78,0.88)
**σ_X**	Residual error standard deviation (intracellular measurements)	1.12 (1.06,1.18)	0.95 (0.90,1.01)	1.07 (1.01,1.14)	0.91 (0.86,0.97)
**L**	Log-likelihood	-1895.97 (-1903.09,-1890.71)	-1663.20 (-1669.97,-1658.09)	-1822.10 (-1830.17,-1816.64)	-1619.87 (-1627.66,-1614.25)
**DIC**	Deviance Information Criterion	3,801	3,340	3,655	3,254

Table F: Posterior Parameter Estimates (RL Strain). Sensitivity of posterior parameter estimates to data excluded during model fitting. Here, measurements below the limit of quantification (crosses in Figure 1) were included during model fitting. Measurements below the LOD were left censored at the LOD during model fitting and we assumed that the antiviral directly inhibits the transition process of infected cells to infectious (virion producing) cells , i.e., acts on τ.

		16681 (No Refresh)			
**Parameter**	**Description**	**Raw Data (no data excluded)**	**16681 Day 4 Included, outliers excluded**	**Outliers included, 16681 day 4 excluded**	**Final Dataset**
**β**	Infection rate of target cells per virion (day^{-1})	$9.26x10^{-09}$ ($6.17x10^{-09}$,$1.40x10^{-08}$)	$9.98x10^{-09}$ ($6.98x10^{-09}$,$1.42x10^{-08}$)	$1.50x10^{-08}$ ($1.04x10^{-08}$,$2.19x10^{-08}$)	$1.32x10^{-08}$ ($9.05x10^{-09}$,$1.88x10^{-08}$)
**ω**	Intracellular virus production rate per infectious cell (day^{-1})	$9.67x10^{6}$ ($6.02x10^{6}$,$1.55x10^{7}$)	$1.05x10^{7}$ ($7.07x10^{6}$,$1.47x10^{7}$)	$1.41x10^{7}$ ($9.28x10^{6}$,$2.36x10^{7}$)	$1.36x10^{7}$ ($9.43x10^{6}$,$1.89x10^{7}$)
**p**	Proportion of intracellular RNA becoming extracellular virus	$3.92x10^{-01}$ ($2.40x10^{-01}$,$6.25x10^{-01}$)	$3.11x10^{-01}$ ($2.09x10^{-01}$,$4.69x10^{-01}$)	$2.10x10^{-01}$ ($1.24x10^{-01}$,$3.19x10^{-01}$)	$2.28x10^{-01}$ ($1.55x10^{-01}$,$3.55x10^{-01}$)
**IC_{50}**	Concentration at which 50% of maximum effect is achieved (nM)	$1.05x10^{-02}$ ($7.16x10^{-03}$,$1.51x10^{-02}$)	$1.54x10^{-02}$ ($1.06x10^{-02}$,$2.09x10^{-02}$)	$6.31x10^{-03}$ ($3.78x10^{-03}$,$9.51x10^{-03}$)	$1.28x10^{-02}$ ($9.00x10^{-03}$,$1.79x10^{-02}$)
**h**	Hill coefficient	0.96 (0.86,1.05)	1.13 (1.02,1.27)	0.91 (0.80,1.01)	1.17 (1.07,1.31)
**R0**	Basic reproduction number	162.43 (137.57,198.75)	151.99 (133.06,178.27)	206.36 (173.08,249.12)	191.10 (161.84,232.76)
**σ_V**	Residual error standard deviation (extracellular measurements)	0.88 (0.82,0.93)	0.82 (0.77,0.87)	0.89 (0.83,0.94)	0.83 (0.78,0.88)
**σ_X**	Residual error standard deviation (intracellular measurements)	1.12 (1.06,1.18)	0.95 (0.90,1.01)	1.07 (1.01,1.14)	0.91 (0.86,0.97)
**L**	Log-likelihood	-1895.97 (-1903.09,-1890.71)	-1663.20 (-1669.97,-1658.09)	-1822.10 (-1830.17,-1816.64)	-1619.87 (-1627.66,-1614.25)
**DIC**	Deviance Information Criterion	3,801	3,340	3,655	3,254

Table G: Posterior Parameter Estimates (16681 Strain). Sensitivity of posterior parameter estimates to data excluded during model fitting. Here, measurements below the limit of quantification (crosses in Figure 1) were included during model fitting. Measurements below the LOD were left censored at the LOD during model fitting and we assumed that the antiviral directly inhibits the transition process of infected cells to infectious (virion producing) cells , i.e., acts on τ.

6 Deriving the basic reproduction number

The basic reproduction number (R_0) for our model is defined as the mean number of infected cells produced by each infected cell over its lifespan at the start of infection and without therapeutic intervention, in an individual well.

In the absence of the antiviral, the dynamics of the cell population in an individual well are described by the following set of equations

$$\frac{dT}{dt} = s_N\, T - k_T\, \left(1 + \frac{N}{K_w}\right)\, T - \beta V\, T$$
$$\frac{dE}{dt} = \beta V T - k_T \left(1 + \frac{N}{K_w}\right) E - k_I E - \tau E \qquad (11)$$
$$\frac{dI}{dt} = \tau E - k_T \left(1 + \frac{N}{K_w}\right) I - k_I I$$
$$\frac{dX}{dt} = \omega\, I - \epsilon\, X$$
$$\frac{dV}{dt} = p\epsilon\, X - k_V\, V$$

where N=T+x10+I.

As the target cell population was not at equilibrium at the start of infection, to calculate R_0, we first need to determine the number of target cells in each well at the start of infection, which we denote T*=T(t*). We have that:

$$\frac{dT}{dt} = s_N T - k_T \left(1 + \frac{T}{K_w}\right)$$
$$= k_T \left(\frac{s_N}{k_T} - 1 - \frac{T}{K_w}\right) T \qquad (12)$$
$$= k_T \left(\hat{s} - \frac{T}{K_w}\right) \mathrm{T}$$

where $\hat{s} = \left(\frac{s_N}{k_T} - 1\right)$. Therefore,

$$\frac{dT}{T\left(\hat{s} - \frac{T}{K_w}\right)} = k_T dt$$
$$\int \frac{dT}{T} + \int \frac{\mathrm{d}T}{\hat{s} K_w - T} = \int \hat{s} k_T \mathrm{dt} \qquad (13)$$
$$ln(T) - ln(\hat{s} K_w - T) = \hat{s} k_T t + C$$

$$\left(\frac{\hat{s}K_w - T}{T}\right) = e^{(-\hat{s}k_T t - C)}$$

$$T(\mathrm{t}) = \frac{\hat{s}K_w}{(1 + \mathrm{A}e^{(-\hat{s}k_T \mathrm{t})})} \quad \text{where} \quad \mathrm{A} = \mathrm{e}^{-\mathrm{C}}$$

$$= \frac{\hat{s}K_w}{1 + \left(\frac{\hat{s}K_w - T_0}{T_0}\right)\mathrm{e}^{(-\hat{s}k_T \mathrm{t})}}$$

where $\mathrm{T}_0 = T(0)$. Hence, given $\hat{s} = \left(\frac{s_N}{k_T} - 1\right)$, we have:

$$\mathrm{T}^* = \mathrm{T}(\mathrm{t}^*) = \frac{K_w(s_N - k_T)T_0}{k_T T_0 + [K_w(s_N - k_T) - k_T T_0]e^{(-(s_N - k_T)t^*)}} \qquad (14)$$

We then use the Next Generation Method to derive the basic reproduction number R_0 for our model[3]. Following this method, R_0 is calculated as the dominant eigenvalue of the matrix FV^{-1} where the matrix F describes the rate of appearance of new infections in each compartment and the matrix V describes the net rate of transfer of individuals out of each compartment. Both F and V are evaluated at the start of infection, when we have a target cell population T^*.

Using equations (11) above, we obtain

$$F = \begin{bmatrix} 0 & 0 & 0 & \beta\mathrm{T}^* \\ 0 & 0 & 0 & 0 \\ 0 & 0 & 0 & 0 \\ 0 & 0 & 0 & 0 \end{bmatrix}, V = \begin{bmatrix} k_I^* + \tau & 0 & 0 & 0 \\ -\tau & k_I^* & 0 & 0 \\ 0 & -\omega & \epsilon & 0 \\ 0 & 0 & -p\epsilon & k_V \end{bmatrix}$$

where $k_I^* = k_T\left(1 + \frac{\mathrm{T}^*}{K_w}\right) + k_I$.

Thus,

$$FV^{-1} = \begin{bmatrix} 0 & 0 & 0 & \beta\mathrm{T}^* \\ 0 & 0 & 0 & 0 \\ 0 & 0 & 0 & 0 \\ 0 & 0 & 0 & 0 \end{bmatrix} \begin{bmatrix} \frac{1}{k_I^* + \tau} & 0 & 0 & 0 \\ \frac{\tau}{k_I^*(k_I^* + \tau)} & \frac{1}{k_I^*} & 0 & 0 \\ \frac{\tau\omega}{k_I^*(k_I^* + \tau)\epsilon} & \frac{\omega}{k_I^*\epsilon} & \frac{1}{\epsilon} & 0 \\ \frac{\tau\omega p}{k_I^*(k_I^* + \tau)k_V} & \frac{\omega p}{k_I^* k_V} & \frac{p}{k_V} & \frac{1}{k_V} \end{bmatrix} = \begin{bmatrix} \frac{\beta\mathrm{T}^*\tau\omega p}{k_I^*(k_I^* + \tau)k_V} & \frac{\beta\mathrm{T}^*\omega p}{k_I^* k_V} & \frac{\beta\mathrm{T}^* p}{k_V} & \frac{\beta\mathrm{T}^*}{k_V} \\ 0 & 0 & 0 & 0 \\ 0 & 0 & 0 & 0 \\ 0 & 0 & 0 & 0 \end{bmatrix}$$

As FV^{-1} is an upper-triangular matrix, its eigenvalues are the diagonal entries of the matrix. Hence, as R_0 is defined as the dominant eigenvalue of this matrix, we obtain

$$R_0 = \frac{\beta T^* \tau \omega p}{k_I^*(k_I^* + \tau) k_V} \quad (15)$$

where $k_I^* = k_T\left(1 + \frac{T^*}{K_w}\right) + k_I$ and

$$T^* = T(t^*) = \frac{K_w(s_N - k_T)T_0}{k_T T_0 + [K_w(s_N - k_T) - k_T T_0] e^{(-(s_N - k_T)t^*)}} \quad (16)$$

The basic reproduction number for the model version without latent stage has been computed using the same rationale presented above, and is given by:

$$R_0 = \frac{\beta T^* \omega p}{k_I^* k_V} \quad (17)$$
