# Supplementary material for: Modelling the impact of JNJ-1802, a first-in-class dengue inhibitor blocking the NS3-NS4B interaction, on in-vitro DENV-2 dynamics
Source: PLoS Comput Biol. 2023 Dec 6;19(12):e1011662. doi: 10.1371/journal.pcbi.1011662 (PMC10699615; doi:10.1371/journal.pcbi.1011662)
Supplement: Attachment — Submitted filename: Response to Reviewers.pdf [file pcbi.1011662.s004.pdf]

Point-by-point response letter

Modelling the impact of JNJ-1802, a first-in-class dengue inhibitor blocking the NS3-NS4B interaction, on in-vitro DENV-2 dynamics

Clare P. McCormack, Olivia Goethals, Nele Goeyvaerts, Xavier Woot de Trixhe, Peggy Geluykens, Doortje Borrenberghs, Neil M. Ferguson, Oliver Ackaert, Ilaria Dorigatti

Reviewer #1:

This is a nicely done analysis on an interesting topic. The paper is well written and clear. I have just a couple of minor comments/suggestions.

Thank you for the positive feedback.

- Introduction:
  Line 72: extra "I" typo

  This has now been corrected.

- With the results before methods order it would be helpful to have a brief description of the model at the start of the results

  We thank the reviewer for this suggestion, and have added the following text on lines 127-132:

  "For both strains, we quantified the impact of JNJ-1802 on infection in Vero cells by fitting a mechanistic viral kinetics model of the dynamics of DENV infection in-vitro simultaneously to the intracellular and extracellular RNA data observed from the experimental infection studies. ***The model describes the infection process of Vero cells and subsequent secretion of viral RNA, and assumes JNJ-1802 acts on the transition process of infected to infectious (virion producing) cells.*** *Full details of the model developed, parameter values, and model fitting approach are provided in the Methods section below."*

- Suggest replacing e with 10 in the numbers.

  We have replaced 'e' with 'x10' where relevant in the main text and supplementary material.

- Line 257: "amounting to" – found this wording a little confusing- suggest rephrasing

  We have rephased this sentence as follows (lines 268-272):

  "To the best of our knowledge, these are the first estimates of DENV viral fitness, as measured by the in-vitro R_0, which not only provide important insight into the transmissibility of these strains, but also allow to quantify the reduction in R_0 required for viral suppression (R_e<1). ***Here we estimate*** *an average* ***reduction in R_0*** *of 99.6% and 99.4%* ***is required*** *for DENV-2/RL and DENV-2/16681 strains, respectively."*

- Discussion:
  Suggest adding something on the timing of administration- even with excellent inhibition what can the impact be if given later in infection?

For the experiments discussed here, JNJ-1802 was added to each well prior to cells being infected and thus we were unable to assess the impact of administering JNJ-1802 after cells are infected. Hence, we are cautious about speculating on the impact of the timing of administration in the absence of *in-vitro* experimental data. However, results from pre-clinical experimental challenge studies which explored the impact of administering JNJ-1802 therapeutically are encouraging as JNJ-1802 was found to be effective in reducing viral load in AG129 mice when treatment with JNJ-1802 was initiated on day 4 post infection with DENV-2 in AG129 mice[1].

Reviewer #2:

The authors of this manuscript present the results of a within-host modeling study in which they sought to characterize the impact of a novel antiviral for dengue (JNJ-1802) using experimental in-vitro DENV-2 data. They find that drug concentrations >1.6 nM induce viral suppression, with similar results across the two strains of DENV-2 used in the experimental studies. This study provides useful information on the profile of JNJ-1802 as well as a potential framework for studying this drug in different host systems and with different dengue serotypes. Furthermore, these results could be used to help inform dosing regimens of the drug. Overall, the authors build a robust model that provides a more clear understanding of JNJ-1802 than can be obtained from the experimental results alone.

Thank you for the positive feedback.

However, there are some points I would like the authors to address, listed below:

1) Lines 97-98: It is unclear exactly what modeling will contribute to our general understanding of the impact of JNJ-1802. Some justification of the approach would help convince the reader of the importance of this study; this is briefly discussed in lines 245-246, but should also be done so here. This is done to some extent in lines 105-107, although it's unclear how much this applies to this drug considering it is already in clinical trials (lines 92-93).

   We have added the following text to the Introduction (lines 98-105):

   "The use of mechanistic models based on ordinary differential equations for reconstructing the within-host dynamics of viral infections in humans [2]–[9]*, including dengue*[4], [10]–[13]*, have been demonstrated to be a powerful tool to help build our understanding of viral kinetics, the host immune response, as well as antiviral drug effects*[14]. ***One of the primary advantages of adopting a mechanistic modelling approach when assessing the impact of JNJ-1802 is that it allows us to make full use of longitudinal viral kinetic data, and this in turn allows us to estimate the antiviral concentration required for viral suppression in Vero cells."***

2) Figure 1 caption: The authors note that they exclude data from day 4 for the intracellular data from strain 16681 due to "inconsistency" with the extracellular data. Do the authors have any hypotheses as to why this data was inconsistent (possibly measurement error)? Why do they assume that this data does not reveal anything about the mechanism of the drug, and can be excluded? How do the findings change if this data is included? I'm also interested in the answer to this last question with regards to the data that was > 1 log10 difference from the median, which was also removed?

   We do not know the exact cause of the inconsistency between the intracellular and extracellular data collected on day 4 for the 16681 strain. However, we believe it is most likely due to

measurement error. For both hypotheses on mode of drug action, we tested the sensitivity of our results to the exclusion of this data and to the exclusion of the data with >1 log10 difference from the median (please see results in Tables 1-2 below). Across all scenarios, the 95% credible intervals for our estimates of the IC_{50}, Hill co-efficient and basic reproduction number were largely overlapping and thus we obtain consistent estimates of the effect profile of JNJ-1802.

We have added the tables provided below the Supplementary Materials (Supplementary Tables S6-S9), and have added the following text to the Results (lines 237-245):

"Intracellular data collected on day 4 for experiments using the DENV-2 16681 strain and JNJ-1802 concentrations of 0 nM, $2.56x10^{-03}$ nM, $1.28x10^{-02}$ nM, and $6.40x10^{-02}$ nM was excluded from the final model fit because this data was inconsistent with the corresponding extracellular data (Figure 1). In addition, measurements with > 1 log_{10} difference from the corresponding median during model fitting were also excluded because they were considered outlying (Figure 1). We tested the sensitivity of our results to the exclusion of this data and found that, across all scenarios, the 95% credible intervals for our posterior estimates of the IC_{50}, Hill co-efficient and R_0 were largely overlapping (*Supplementary Tables S6,S7*) *and thus we obtain consistent estimates of the in-vitro prophylactic inhibitory effect profile of JNJ-1802."*

		RL Strain (No Refresh)			
**Parameter**	**Description**	**Raw Data (no data exluded)**	**16681 Day 4 Included, outliers excluded**	**Outliers included, 16681 day 4 excluded**	**Final Dataset**
**β**	Infection rate of target cells per virion (day^{-1})	2.96E-08 (2.06E-08,4.22E-08)	3.01E-08 (2.21E-08,4.44E-08)	2.99E-08 (2.01E-08,4.39E-08)	3.18E-08 (2.29E-08,4.40E-08)
**p**	Proportion of intracellular RNA becoming extracellular virus	6.45E-01 (4.10E-01,9.36E-01)	5.75E-01 (3.89E-01,8.57E-01)	6.12E-01 (3.88E-01,9.10E-01)	5.90E-01 (3.99E-01,8.83E-01)
**ω**	Intracellular virus production rate per infectious cell (day^{-1})	3.14E+06 (2.07E+06,4.42E+06)	3.32E+06 (2.37E+06,4.81E+06)	3.19E+06 (2.10E+06,4.59E+06)	3.17E+06 (2.32E+06,4.44E+06)
**IC_{50}**	Concentration at which 50% of maximum effect is achieved (nM)	9.06E-03 (5.68E-03,1.33E-02)	1.24E-02 (6.87E-03,1.75E-02)	8.79E-03 (5.58E-03,1.51E-02)	1.23E-02 (8.33E-03,1.66E-02)
**h**	Hill coefficient	1.08 (0.96,1.21)	1.28 (1.10,1.45)	1.07 (0.95,1.24)	1.28 (1.14,1.42)
**$σ_v$**	Residual error standard deviation (extracellular measurements)	0.88 (0.82,0.93)	0.82 (0.77,0.87)	0.89 (0.83,0.94)	0.83 (0.78,0.88)
**$σ_X$**	Residual error standard deviation (intracellular measurements)	1.12 (1.06,1.18)	0.95 (0.90,1.01)	1.07 (1.01,1.14)	0.91 (0.86,0.97)
**L**	Log-likelihood	-1895.97 (-1903.09,-1890.71)	-1663.20 (-1669.97,-1658.09)	-1822.10 (-1830.17,-1816.64)	-1619.87 (-1627.66,-1614.25)
**R_0**	Basic reproduction number	270.57 (225.55,330.09)	273.14 (227.57,335.63)	269.57 (219.44,328.39)	277.01 (231.78,332.66)
**DIC**	Deviance Information Criterion	3,801	3,340	3,655	3,254

Table 1: The sensitivity of posterior parameter estimates to data excluded during model fitting for the DENV-2/RL strain, with no medium refresh. Here, we assume the antiviral directly inhibits the transition process of infected cells to infectious (virion producing) cells , i.e., acts on τ.

		16681 Strain (No Refresh)			
Parameter	Description	Raw Data (no data exluded)	16681 Day 4 Included, outliers excluded	Outliers included, 16681 day 4 excluded	Final Dataset
**β**	Infection rate of target cells per virion (day^{-1})	9.26E-09 (6.17E-09,1.40E-08)	9.98E-09 (6.98E-09,1.42E-08)	1.50E-08 (1.04E-08,2.19E-08)	1.32E-08 (9.05E-09,1.88E-08)
**p**	Proportion of intracellular RNA becoming extracellular virus	3.92E-01 (2.40E-01,6.25E-01)	3.11E-01 (2.09E-01,4.69E-01)	2.10E-01 (1.24E-01,3.19E-01)	2.28E-01 (1.55E-01,3.55E-01)
**ω**	Intracellular virus production rate per infectious cell (day^{-1})	9.67E+06 (6.02E+06,1.55E+07)	1.05E+07 (7.07E+06,1.47E+07)	1.41E+07 (9.28E+06,2.36E+07)	1.36E+07 (9.43E+06,1.89E+07)
**IC_{50}**	Concentration at which 50% of maximum effect is achieved (nM)	1.05E-02 (7.16E-03,1.51E-02)	1.54E-02 (1.06E-02,2.09E-02)	6.31E-03 (3.78E-03,9.51E-03)	1.28E-02 (9.00E-03,1.79E-02)
**h**	Hill coefficient	0.96 (0.86,1.05)	1.13 (1.02,1.27)	0.91 (0.80,1.01)	1.17 (1.07,1.31)
**σ_v**	Residual error standard deviation (extracellular measurements)	0.88 (0.82,0.93)	0.82 (0.77,0.87)	0.89 (0.83,0.94)	0.83 (0.78,0.88)
**σ_X**	Residual error standard deviation (intracellular measurements)	1.12 (1.06,1.18)	0.95 (0.90,1.01)	1.07 (1.01,1.14)	0.91 (0.86,0.97)
**L**	Log-likelihood	-1895.97 (-1903.09,-1890.71)	-1663.20 (-1669.97,-1658.09)	-1822.10 (-1830.17,-1816.64)	-1619.87 (-1627.66,-1614.25)
**R_0**	Basic reproduction number	162.43 (137.57,198.75)	151.99 (133.06,178.27)	206.36 (173.08,249.12)	191.10 (161.84,232.76)
**DIC**	Deviance Information Criterion	3,801	3,340	3,655	3,254

Table 2: The sensitivity of posterior parameter estimates to data excluded during model fitting for the DENV-2/16681 strain, with no medium refresh. Here, we assume the antiviral directly inhibits the transition process of infected cells to infectious (virion producing) cells , i.e., acts on τ.

3) Figure 1 caption continued: Please keep the y-axis limits the same for all panels (they are slightly misaligned). Also, it is not explained what the triangles in the figures represent. In line 707, please correct “red squares” to “squares”.

We thank the reviewer for drawing this to our attention. The figure and figure legend have been amended as suggested (please see below).

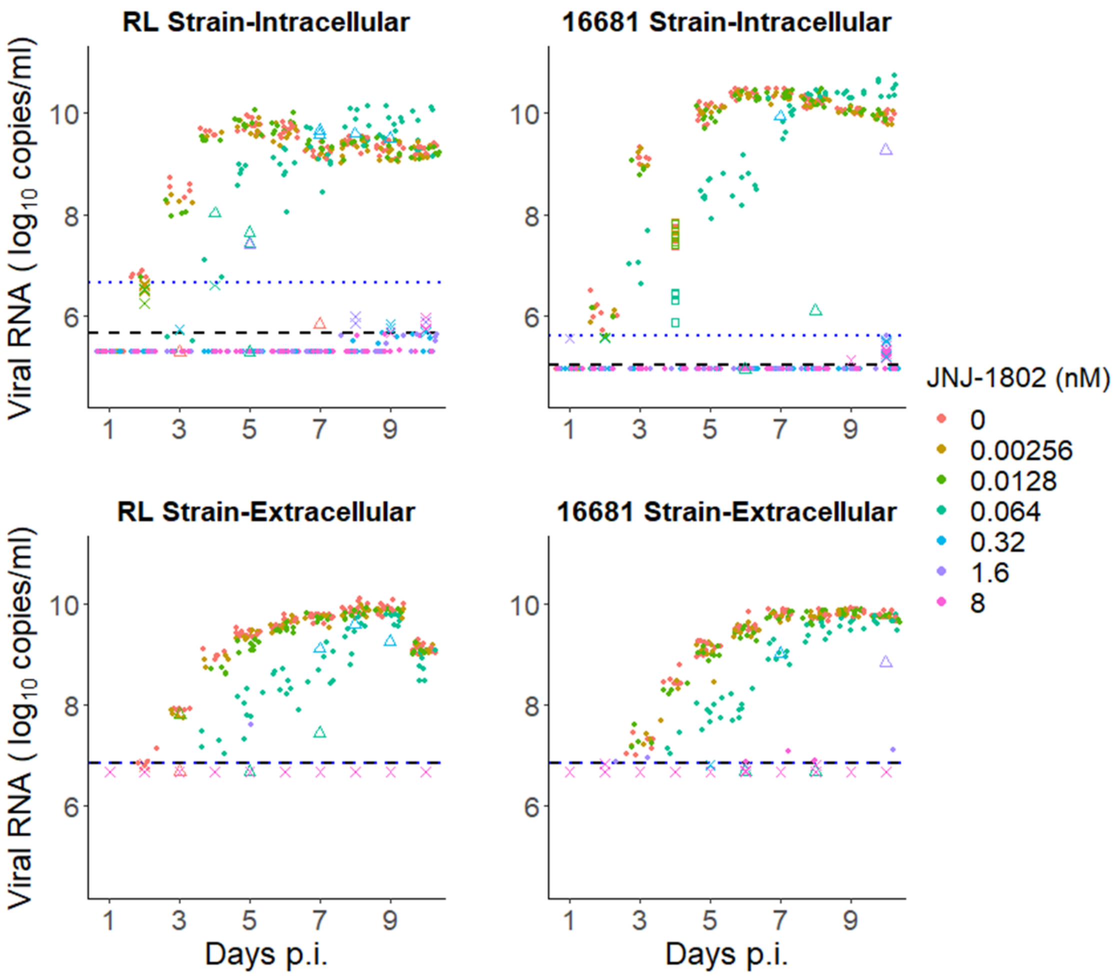

Figure 1: Summary of viral load data from the in-vitro infection experiments in Vero cells. Summary of all data collected, disaggregated by the infecting DENV-2 strain (RL, 16681), JNJ-1802 concentration and type of viral RNA (intra- or extracellular). Measurements with > 1 $\log_{10}$ difference from the corresponding median (**triangles**) were not used for model fitting. We also excluded the intracellular data collected on day 4 for experiments using the DENV-2/16681 dengue strain and antiviral concentrations of 0 nM, $2.56\text{x}10^{-03}$ nM, $1.28\text{x}10^{-02}$ nM, and $6.40\text{x}10^{-02}$ nM, as these were inconsistent with the corresponding extracellular data (18 measurements in total, **squares** in subfigure B). The limit of detection (LOD) and the limit of quantification (LOQ) are indicated by horizontal black and blue lines, respectively. For extracellular data, these limits were identical. Crosses represent measurements below the limit of quantification which were assumed to be left censored in a sensitivity analysis.

4) Lines 164-168, line 254: Why do the two viral strains have different fitness? Are these strains only used in experiments or are either of them endemic; if so, where? Additional context on these strains, and potential implications for their differences could be expanded upon in the discussion.

The two DENV-2 strains used in the experiments conducted in this manuscript are genetically different, as DENV-2/16681 is a G5/Asian I and DENV-2/RL is a G1/American strain according to the Genotype Detective Dengue Virus Typing Tool. As a result, they can also differ in fitness and pathogenicity. Both strains are extensively used for laboratory studies. DENV-2/16681 is cultured starting from a plasmid, and further details on the origins of these strains is provided in previously published work by Kaptein et. al [15] and Goethals et al [1]. These references have been cited in the Methods section of the manuscript (line 361), in the Introduction (lines 89-96), and in the Discussion (lines 253, 277-283, 306,318).

5) Line 310: The fact that this study can be used to inform dosing is important, and could be briefly mentioned in the introduction as additional justification for performing the study.

We have added the following to the Introduction (lines 109-110):

*"This work highlights how interdisciplinary research and mathematical modelling applied to pre-clinical data can help identify promising drug candidates from the early phases of drug development **and help inform dosing regimens**."*

6) Table 3: Uninformative priors can produce unreliable results, as detailed by Lemoine, 2019: https://onlinelibrary.wiley.com/doi/full/10.1111/oik.05985. Uniform distributions are not always a good choice; it may be wise to perform a prior predictive check or to alter the priors used.

We thank the reviewer for this suggestion, and have tested the sensitivity of our results to the choice of prior distributions by re-running the analysis using the following more informative prior distributions:

Symbol	Parameter Description	Uniform Priors	Weakly Informative Priors
β	Target cell infection rate (per virion day^{-1})	$\log_{10}(\beta)$~U[-12,2]	$\log_{10}(\beta)$~Normal(-5,3)
ω	Intracellular RNA synthesis rate (per infectious cell, day^{-1})	$\log_{10}(\omega)\sim$U[-6,10]	$\log_{10}(\omega)$~Normal(1,4)
p	Proportion of intracellular RNA becoming extracellular RNA	$\log_{10}(p)$~U[-6,0]	$\log_{10}(p)$~Normal(-3,2)
IC_{50}	Concentration which achieves 50% of maximum effect	$\log_{10}(IC_{50})$~U[-6,0]	$\log_{10}(IC_{50})$~Normal(-3,2)
h	Hill coefficient	$\log_{10}(h)$~U[-6,1]	$\log_{10}(h)$~Normal(0,2)
σ_X	Residual error standard deviation (Intracellular measurements on $\log_{10}$ scale)	$\log_{10}(\sigma_X)$~U[-6,1]	$\log_{10}(\sigma_X)$~Normal(0,2)
σ_V	Residual error standard deviation (Extracellular measurements on $\log_{10}$ scale)	$\log_{10}(\sigma_V)$~U[-6,1]	$\log_{10}(\sigma_V)$~Normal(0,2)

We found that despite some minor shifts in the central estimates of some parameters, the 95% credible intervals overlap (please see Table 3 below), which indicates that there is sufficient power to estimate the effect profile of JNJ-1802 even in the absence of prior information.

		RL Strain (No Refresh)		16681 Strain (No Refresh)	
Parameter	Description	Weakly Informative Priors	Uniform Priors	Weakly Informative Priors	Uniform Priors
β	Infection rate of target cells per virion (day^{-1})	4.15E-08 (2.90E-08,6.09E-08)	3.18E-08 (2.29E-08,4.40E-08)	1.76E-08 (1.22E-08,2.60E-08)	1.32E-08 (9.05E-09,1.88E-08)
p	Proportion of intracellular RNA becoming extracellular virus	3.73E-01 (2.36E-01,5.76E-01)	5.90E-01 (3.99E-01,8.83E-01)	1.67E-01 (1.17E-01,2.39E-01)	2.28E-01 (1.55E-01,3.55E-01)
ω	Intracellular virus production rate per infectious cell (day^{-1})	3.39E+06 (2.50E+06,4.71E+06)	3.17E+06 (2.32E+06,4.44E+06)	1.29E+07 (9.09E+06,1.85E+07)	1.36E+07 (9.43E+06,1.89E+07)
IC_{50}	Concentration at which 50% of maximum effect is achieved (nM)	1.43E-02 (1.02E-02,2.03E-02)	1.23E-02 (8.33E-03,1.66E-02)	1.59E-02 (1.15E-02,2.21E-02)	1.28E-02 (9.00E-03,1.79E-02)
h	Hill coefficient	1.33 (1.20,1.50)	1.28 (1.14,1.42)	1.27 (1.15,1.43)	1.17 (1.07,1.31)
$σ_v$	Residual error standard deviation (extracellular measurements)	0.84 (0.79,0.90)	0.83 (0.78,0.88)	0.84 (0.79,0.90)	0.83 (0.78,0.88)
$σ_x$	Residual error standard deviation (intracellular measurements)	0.91 (0.86,0.96)	0.91 (0.86,0.97)	0.91 (0.86,0.96)	0.91 (0.86,0.97)
L	Log-likelihood	-1630.68 (-1644.00,-1621.49)	-1619.87 (-1627.66,-1614.25)	-1630.68 (-1644.00,-1621.49)	-1619.87 (-1627.66,-1614.25)
R_0	Basic reproduction number	248.72 (205.42,294.99)	277.01 (231.78,332.66)	182.19 (154.89,214.04)	191.10 (161.84,232.76)
DIC	Deviance Information Criterion	3,277	3,254	3,277	3,254

Table 3: The sensitivity of posterior parameter estimates to the choice of prior distribution during for the experiments with no medium refresh.

7) In the discussion, it should be noted that one of the limitations of the study is that quite a few of the parameter values were fixed (and some were not informed directly by literature), rather than all of the parameters being fit to the data.

We have added the following text to the Discussion (lines 284-286):

*"An additional limitation of our study is **that several model parameters were fixed, including those governing the underlying cell dynamics.** We also did not have an exact measurement of infectious virus at the start of infection (which is below the limit of detection)..."*

References

[1] O. Goethals *et al.*, "Blocking NS3–NS4B interaction inhibits dengue virus in non-human primates," *Nature*, pp. 1–9, Mar. 2023.

[2] C. A. A. Beauchemin and A. Handel, "A review of mathematical models of influenza A infections within a host or cell culture: Lessons learned and challenges ahead," *BMC Public Health*, vol. 11, no. SUPPL. 1, 2011.

[3] A. Handel, I. M. Longini, and R. Antia, "Towards a quantitative understanding of the within-host dynamics of influenza A infections," *J. R. Soc. Interface*, vol. 7, no. 42, pp. 35–47, Jun. 2009.

[4] R. Ben-Shachar, S. Schmidler, and K. Koelle, "Drivers of Inter-individual Variation in Dengue Viral Load Dynamics," *PLoS Comput. Biol.*, vol. 12, no. 11, p. 1005194, Nov. 2016.

[5] A. W. C. Yan, J. Zhou, C. A. A. Beauchemin, C. A. Russell, W. S. Barclay, and S. Riley, "Quantifying mechanistic traits of influenza viral dynamics using in vitro data," *Epidemics*, vol.

33, p. 100406, Dec. 2020.

[6] K. Koelle, A. P. Farrell, C. B. Brooke, and R. Ke, "Within-host infectious disease models accommodating cellular coinfection, with an application to influenza," *Virus Evol.*, vol. 5, no. 2, p. 18, Jul. 2019.

[7] A. S. Perelson and R. M. Ribeiro, "Modeling the within-host dynamics of HIV infection," *BMC Biology*, vol. 11, no. 1. BioMed Central, pp. 1–10, 03-Sep-2013.

[8] A. Goyal, L. E. Liao, and A. S. Perelson, "Within-host mathematical models of hepatitis B virus infection: Past, present, and future," *Curr. Opin. Syst. Biol.*, vol. 18, pp. 27–35, Dec. 2019.

[9] A. U. Neumann *et al.*, "Hepatitis C viral dynamics in vivo and the antiviral efficacy of interferon-α therapy," *Science*, vol. 282, no. 5386, pp. 103–107, Oct. 1998.

[10] H. E. Clapham, V. Tricou, N. Van Vinh Chau, C. P. Simmons, and N. M. Ferguson, "Within-host viral dynamics of dengue serotype 1 infection," *J. R. Soc. Interface*, vol. 11, no. 96, p. 20140094, Jul. 2014.

[11] H. E. Clapham, T. H. Quyen, D. T. H. Kien, I. Dorigatti, C. P. Simmons, and N. M. Ferguson, "Modelling Virus and Antibody Dynamics during Dengue Virus Infection Suggests a Role for Antibody in Virus Clearance," *PLoS Comput. Biol.*, vol. 12, no. 5, p. e1004951, May 2016.

[12] R. Ben-Shachar and K. Koelle, "Minimal within-host dengue models highlight the specific roles of the immune response in primary and secondary dengue infections," *J. R. Soc. Interface*, vol. 12, no. 103, 2015.

[13] N. Nuraini, H. Tasman, E. Soewono, and K. A. Sidarto, "A with-in host Dengue infection model with immune response," *Math. Comput. Model.*, vol. 49, no. 5–6, pp. 1148–1155, Mar. 2009.

[14] L. Canini and A. S. Perelson, "Viral kinetic modeling: State of the art," *J. Pharmacokinet. Pharmacodyn.*, vol. 41, no. 5, pp. 431–443, Oct. 2014.

[15] S. J. F. F. Kaptein *et al.*, "A pan-serotype dengue virus inhibitor targeting the NS3–NS4B interaction," *Nature*, vol. 598, no. 7881, pp. 504–509, Oct. 2021.
