# Supplementary material for: Modelling the impact of JNJ-1802, a first-in-class dengue inhibitor blocking the NS3-NS4B interaction, on in-vitro DENV-2 dynamics
Source: PLoS Comput Biol. 2023 Dec 6;19(12):e1011662. doi: 10.1371/journal.pcbi.1011662 (PMC10699615; doi:10.1371/journal.pcbi.1011662)
Supplement: Attachment — Submitted filename: PCB_McCormack2023.docx [file pcbi.1011662.s003.docx]

The authors of this manuscript present the results of a within-host modeling study in which they sought to characterize the impact of a novel antiviral for dengue (JNJ-1802) using experimental in-vitro DENV-2 data. They find that drug concentrations >1.6 nM induce viral suppression, with similar results across the two strains of DENV-2 used in the experimental studies. This study provides useful information on the profile of JNJ-1802 as well as a potential framework for studying this drug in different host systems and with different dengue serotypes. Furthermore, these results could be used to help inform dosing regimens of the drug. Overall, the authors build a robust model that provides a more clear understanding of JNJ-1802 than can be obtained from the experimental results alone.

However, there are some points I would like the authors to address, listed below:

1) Lines 97-98: It is unclear exactly what modeling will contribute to our general understanding of the impact of JNJ-1802. Some justification of the approach would help convince the reader of the importance of this study; this is briefly discussed in lines 245-246, but should also be done so here. This is done to some extent in lines 105-107, although it’s unclear how much this applies to this drug considering it is already in clinical trials (lines 92-93).
2) Figure 1 caption: The authors note that they exclude data from day 4 for the intracellular data from strain 16681 due to “inconsistency” with the extracellular data. Do the authors have any hypotheses as to why this data was inconsistent (possibly measurement error)? Why do they assume that this data does not reveal anything about the mechanism of the drug, and can be excluded? How do the findings change if this data is included? I’m also interested in the answer to this last question with regards to the data that was > 1 log10 difference from the median, which was also removed?
3) Figure 1 caption continued: Please keep the y-axis limits the same for all panels (they are slightly misaligned). Also, it is not explained what the triangles in the figures represent. In line 707, please correct “red squares” to “squares”.
4) Lines 164-168, line 254: Why do the two viral strains have different fitness? Are these strains only used in experiments or are either of them endemic; if so, where? Additional context on these strains, and potential implications for their differences could be expanded upon in the discussion.
5) Line 310: The fact that this study can be used to inform dosing is important, and could be briefly mentioned in the introduction as additional justification for performing the study.
6) Table 3: Uninformative priors can produce unreliable results, as detailed by Lemoine, 2019: <https://onlinelibrary.wiley.com/doi/full/10.1111/oik.05985>. Uniform distributions are not always a good choice; it may be wise to perform a prior predictive check or to alter the priors used.
7) In the discussion, it should be noted that one of the limitations of the study is that quite a few of the parameter values were fixed (and some were not informed directly by literature), rather than all of the parameters being fit to the data.
